# Land use, REDD+ and the status of wildlife populations in Yaeda Valley, northern Tanzania

**DOI:** 10.1371/journal.pone.0214823

**Published:** 2019-04-04

**Authors:** Christian Kiffner, Zoe Arndt, Trent Foky, Megan Gaeth, Alex Gannett, Madeline Jackson, Georgie Lellman, Sophia Love, Ana Maroldi, Shane McLaughlin, Bobbi Skenandore, Sarah von Euler, Zachary Zambrano, Bernard Kissui

**Affiliations:** 1 Center For Wildlife Management Studies, The School For Field Studies, Karatu, Tanzania; 2 Zoology Department, Colorado State University, Fort Collins, CO, United States of America; 3 Whitman College, Walla Walla, WA, United States of America; 4 Biology Department, Guilford College, Greensboro, North Carolina, United States of America; 5 Environmental Studies, Gonzaga University, Spokane, WA, United States of America; 6 Ecology, Evolution and Behavior, University of Minnesota, St. Paul, MN, United States of America; 7 Kenyon College, Gambier, OH, United States of America; 8 Department of Veterinary and Animal Science, University of Massachusetts-Amherst, Amherst, MA, United States of America; 9 Environmental Science Program, Trinity College, Hartford, CT, United States of America; 10 Nelson Institute for Environmental Studies, University of Wisconsin-Madison, Madison, Wisconsin, United States of America; 11 Biology Department, Davidson College, Davidson, NC, United States of America; 12 Department of Animal Sciences and Industry, Kansas State University, Manhattan, KS, United States of America; University of South Carolina, UNITED STATES

## Abstract

REDD+ projects primarily focus on reducing carbon emissions from deforestation and forest degradation in developing countries. These projects are regularly evaluated against their core objective of conserving carbon stocks, but their contribution to biodiversity conservation has rarely been assessed. To assess the conservation value of the area and the relative performance of a REDD+ land use plan in Yaeda Valley, a semi-arid savannah ecosystem in northern Tanzania, we implemented an annual wildlife monitoring scheme. Based on direct sightings and indirect signs of wildlife, obtained from stratified walking transects conducted annually from 2015–2018, we estimated annual trends of mammal species richness and wildlife densities in three REDD+ and three non-REDD+ land-use strata. Our surveys document a near complete mammal community in the area. Species accumulation curves, and subsequent statistical comparisons, indicated highest mammal species richness in the woodland habitats (both REDD+ and non REDD+ strata) as compared to more human and livestock impacted areas, and suggested constant species richness from 2015–2018. To estimate stratum- and year-specific livestock and wildlife densities (cattle, donkey, goat and sheep combined, Thomson’s gazelle, Kirk’s dik-dik) and wildlife sign densities (aardvark, bushbuck, bushpig, Kirk’s dik dik, eland, elephant, Maasai giraffe, greater kudu, hyena, impala, lesser kudu, warthog, wildebeest, Plains zebra), we fitted species-specific detection functions in a distance sampling framework. Species-specific densities varied between 2015 and 2018 and showed substantial increases and occasional declines in other species-stratum combinations. However, population growth rates were not systematically associated with specific land-use strata. Although our results do not explicitly provide evidence that REDD+ land-use plans directly co-benefit wildlife conservation, they show that REDD+ areas have the potential to maintain intact wildlife assemblages. To ensure effective long-term conservation outcomes, we advocate for a more formal integration of wildlife conservation goals in the REDD+ scheme.

## Introduction

Worldwide, habitat loss, habitat fragmentation, homogenization of ecosystems, invasive organisms, climate change, and direct exploitation cause reductions of most wild animal populations and succeeding (local) extinctions of species [1–5]. Among mammals, both large herbivores and carnivores are declining in most parts of the world [2,6–8] with East Africa closely mirroring this global trend [9,10]. Yet, East Africa still supports an impressive variety of large mammal populations and substantial wildlife assemblages persist outside fully protected areas, mainly in semi-arid rangelands [11–15]. Despite their critical role for many wildlife species as migratory and seasonal ranges, as well as permanent habitat [16–20], and associated ecosystem services provided by resident and migratory species [2], these rangelands often lose productivity, decline in size (often due to expanding subsistence agriculture and other human-caused land use changes), and experience accelerated rates of wildlife-livestock competition and other forms of conflict between humans and wildlife [21–26].

To strengthen wildlife conservation, Tanzania considerably increased the size of fully protected national parks in the last 20 years [27], but given the current human population growth rate and ensuing demand for space it is unlikely that fully protected areas can be substantially expanded in size in the future. Therefore, effectively conserving wildlife in increasingly human-dominated landscapes requires identification and implementation of conservation and land-use models that mitigate anthropogenic effects while allowing sustainable natural resource utilization by humans [28,29]. In Tanzania, these multiple-use areas include state-run game controlled areas, forest reserves, nature reserves, and game reserves [27], as well as community-based wildlife management areas [30,31]. A rather novel community-based approach of protecting potential wildlife habitat is the REDD+ scheme (“Reducing emissions from deforestation and forest degradation and the role of conservation, sustainable management of forests and enhancement of forest carbon stocks in developing countries”) which was developed by the United Nations Framework Convention on Climate Change (UNFCCC). Under this scheme, carbon offsets generated by avoided deforestation are sold to local and international buyers, and revenue is distributed among participating communities. The incentive based program is conditional, i.e. payments are only distributed if carbon stocks are monitored and conserved effectively [32]. Although focused on protecting forest resources and forest management, an added “co-benefit” of preserving forests may [33], or may not [34], be the conservation of wildlife species.

Assessing or comparing this co-benefit of specific land-uses and land management schemes across space or time requires implementing effective monitoring schemes which allow estimation of species richness and population trends [12,35–38]. Unfortunately, this crucial monitoring component is often missing in community-based conservation schemes [39,40]. Since baseline estimates before conservation implementations are often missing (and before-after control-impact studies hence not feasible), assessments of conservation approaches often rely on spatial comparisons and ideally use the rate of change in species richness or population density as metrics to gauge conservation effectiveness [12,41,42].

To assess how different land-uses and the REDD+ conservation approach affect wildlife distribution, species richness and population trends over time, we monitored wildlife species richness and relative densities by walking transects in three land-use strata inside, and three land-use strata outside, a REDD+ driven land-use plan in Yaeda Valley, northern Tanzania. Walking transects allow systematic distribution of transects and therefore reduce design-based biases of inferred density estimates, but are relatively labor- and cost-intensive [43]. Additionally, walking transects provide an opportunity to incorporate local people in monitoring activities [44,45] and permits assessing direct and indirect signs of animal presence. Recording indirect signs of wildlife can be an effective method to estimate species richness and relative densities, especially if animal signs can be reliably identified by incorporating indigenous knowledge during field work [46–48].

Here we provide baseline information on the value of different land-use and conservation forms in Yaeda Valley for wildlife conservation in northern Tanzania. First, we assessed overall mammal species richness, a crucial state variable for assessing biodiversity [49,50], and evaluated the completeness of the mammal community against an expected mammal species list derived from current distribution maps [51]. Secondly, and more specifically, we estimated trends of species richness and relative densities of mammalian wildlife species over time [49,52,53] within the six distinct sampled habitat strata (three strata under a REDD+ land-use plan and three others without a specific land-use plan). We hypothesized that areas within the REDD+ land-use plan would be characterized by stable wildlife population communities and show stable or increasing trends in population density and that wildlife populations in areas outside the REDD+ land-use plan would be more likely to decline. In addition, we estimated trends of livestock populations because this allowed us to assess if the land-use policies were effectively implemented, and if livestock populations–which potentially affect wildlife distribution and density [14,54,55]–changed over time.

## Methods

### Study area

Yaeda Valley is located in the Mbulu district of Tanzania, south-east of Lake Eyasi and Ngorongoro Conservation Area [56,57]. This semi-arid region experiences three distinct seasons; the short rains from November—December, the long rains from February—May, and the dry season from June—October. On average, the region receives 450 mm of rain per year with a mean monthly temperature range of 25–30°C [58]. The area is mainly inhabited by agro-pastoralist (Iraqw, Isanzu, Nyiramba) and pastoralist (Datoga) ethnicities, and forms the core distribution area of the Hadza, a hunter-gatherer ethnicity [59]. Carbon Tanzania, a Tanzania based social enterprise, has implemented a project within the REDD+ framework in Domanga and Mongo Wa Mono village lands. The REDD+ project has been designed under the *Plan Vivo Standard* which supports local stakeholders, particularly pastoralist Datoga and hunter gatherer Hadza in natural resource management [60,61].

**Fig 1:**
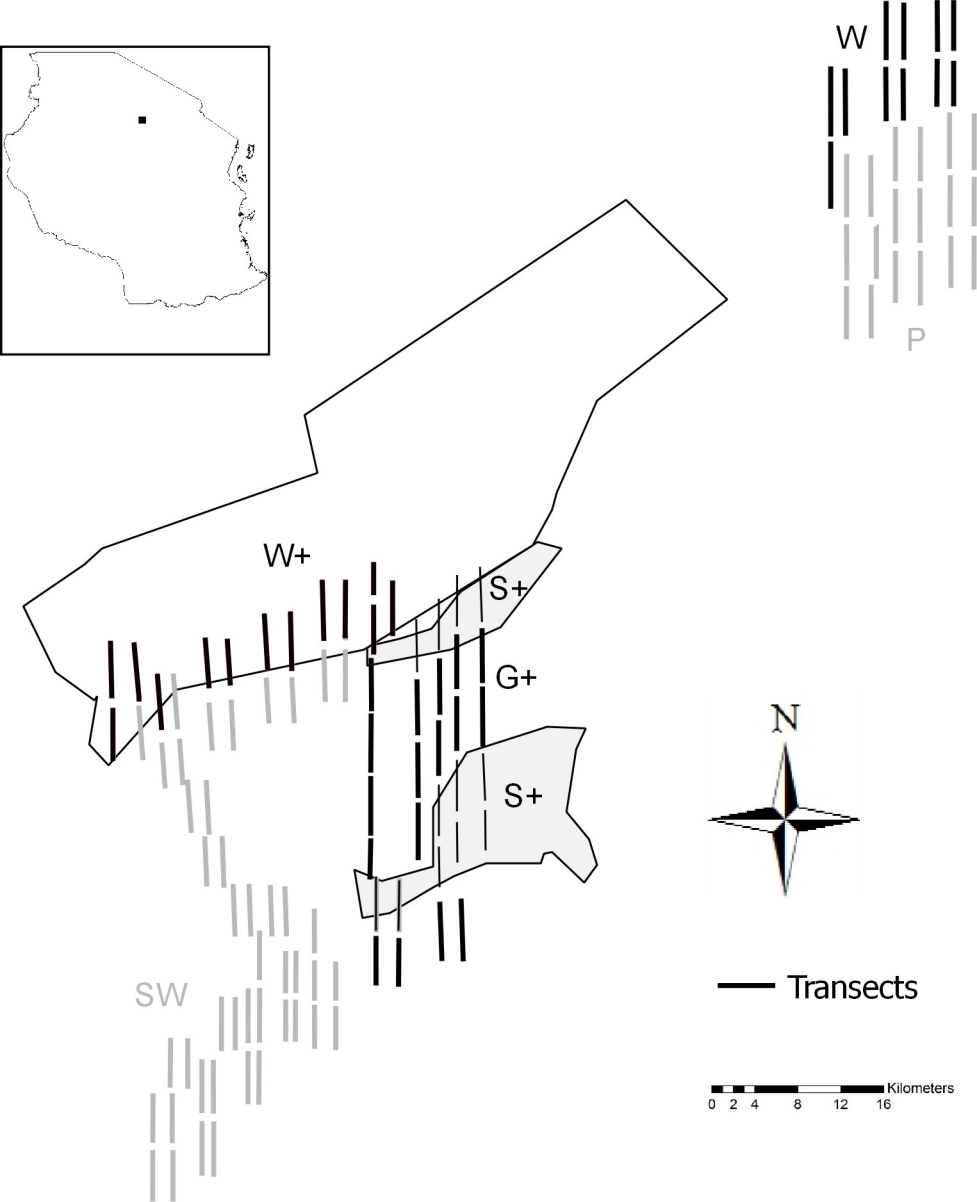
Map of Yaeda Valley and delineated land-use strata. The map shows the distribution of the walking transects in three land use strata defined in the REDD+ scheme [Woodland (W+), Grazing (G+), Settlement (S+)] and three non-REDD+ strata (Woodland (W), Plains (P), and Southern woodland (SW)]. The inset on the top left indicates the location of Yaeda Valley in Tanzania. For clarity, transects were bolded and are hence not to scale.

This study was conducted in six different strata that were delineated according to main vegetation cover and whether or not they were part of the REDD+ land-use plan (Fig 1). Stratification was initially based on main vegetation forms only, and was post-stratified according to management regimes for this analysis. Hence, a few transects dissect multiple land-use strata. In these cases we assigned transects to specific land-use strata if ≥ 50% of the transect fell in the corresponding stratum. Stratified sampling allowed direct assessments of spatial differences in the state, and temporal trends of species richness and (relative) densities of wildlife species.

The three sampled land-use strata that were defined in the REDD+ scheme were Woodland (W+), Grazing (G+), and Settlement (S+). W+ is located in the northwest of the valley along the Kideru ridge, and is dominated by baobab (*Adansonia digitata*) and acacia (*Vachellia seyal* and *Senegalia brevispica*) woodland, interspersed with bushland. The Hadza mainly utilize this area for hunting and foraging. Livestock keeping is not allowed in this area. S+ and G+ are located in the Kideru plains, in the central part of the valley. G+ is designated for livestock grazing and mainly consists of grasslands, seasonal swamps, and acacia woodland. Settlements are not allowed in this area. Areas designated for settlement (S+) are mainly located at slightly higher elevations than the floodplain. Here, settlements, subsistence agriculture and woodland remnants are the main landscape features, and livestock keeping is allowed.

For the control (non-REDD+) strata, we selected three land-use forms that are similar in vegetation structure to the REDD+ land-use strata. W, located in the northwestern corner of our study area, consists of baobab and acacia woodlands on the slopes of the Kideru ridge; this area is also traditionally used by Hadza. The plains (P) to the east of W are also seasonally flooded and constitute a mix of grassland, bushland, and acacia woodland. The Southern woodland (SW), adjacent to W+, G+ and S+, is characterized by relatively flat terrain, a mix of acacia woodland and grasslands and contains small interspersed patches of agriculture established by residents of agro-pastoralist ethnicities. Settlements and livestock keeping occur in this area as well.

### Field sampling

In 2015, we established systematic walking transects using the existing roads as start and endpoints. The same set of 115 transects was completed across all six strata in the short rains (early November) of 2015, 2016, 2017, and 2018. The total effort was 880.3 km (few transects were slightly shorter than 2 km), consisting of 13 transects in W+, 17 in G+, 12 in S+, 11 in W, 18 in P, and 44 in SW (Fig 1). We walked transects in the north-south or south-north direction for 2 km, and we separated consecutive transects by 500 m (north-south). The east-west distance between parallel transects was 1 km to avoid double counting of animals (Fig 1). We used a compass and handheld GPS unit with pre-determined start- and endpoints for bearings and orientation in the field. Three people walked each transect (6 teams per day), with one person being either a village game scout (VGS) or employee of the Mbulu District Game Office. All guides were long-term residents of the study area (from Isanzu, Datoga and Hadza ethnicities) and were knowledgeable in local fauna and sign identification due to formal training and traditional knowledge. Each year we spent the first day training all survey participants in field orientation, sign identification (facilitated by a species list in three languages: English, Swahili, Hadza) and consistent data measurement and recording. Upon seeing a mammal or mammal sign, we identified and recorded detections to species level. In a few cases, unambiguous identification to species level was not possible; therefore we combined closely related species to one species name (e.g. both hyena species, all three potentially occurring hyrax and jackal species, and solitary, similarly-sized mongoose species; Table 1). For each sighting, we recorded the GPS coordinates, cluster size (either herd size or aggregation of animal signs), and perpendicular distances between transect and sighting. Perpendicular distances to animals were measured using a laser range finder (Bushnell Elite 1500), and perpendicular distances to signs were measured using a measuring tape.

**Table 1:** Detected mammal species in Yaeda Valley during walking transects conducted from 2015–2018. Mammal species presence (assessed via signs and/or direct sightings) in the three REDD+ (W+, G+, and S+) and control land-use strata (W, P, and SW) of the Yaeda Valley in 2015 (´15), 2016 (´16), 2017 (´17), and 2018 (´18). Wildlife species expected to be present but not observed are included at the bottom; completeness of the mammal community was calculated as the proportion of medium-large sized mammal species expected to occur in the area. We estimated sign or animal densities for species highlighted in bold only.

		W+				G+				S+				W				P				SW			
Common	Scientific	´15	´16	´17	´18	´15	´16	´17	´18	´15	´16	´17	´18	´15	´16	´17	´18	´15	´16	´17	´18	´15	´16	´17	´18
*Livestock*																									
**Cattle**	*Bos taurus*	**X**	**X**	**X**	**X**	**X**	**X**	**X**	**X**	**X**	**X**	**X**	**X**	**X**		**X**		**X**	**X**	**X**	**X**	**X**	**X**	**X**	**X**
Domestic dog	*Canis lupus familiaris*		X	X		X	X	X	X	X	X	X	X	X				X	X	X	X	X	X	X	X
**Donkey**	*Equus africanus asinus*					**X**	**X**	**X**	**X**	**X**	**X**	**X**	**X**	**X**		**X**		**X**	**X**	**X**	**X**	**X**	**X**	**X**	**X**
Domestic cat	*Felis catus*																							X	X
**Sheep & goat**	*Ovis aries & Capra aegagrus hircus*		**X**			**X**	**X**	**X**	**X**	**X**	**X**	**X**	**X**	**X**				**X**	**X**	**X**	**X**	**X**	**X**	**X**	**X**
*Wildlife*																									
Cheetah	*Acinonyx jubatus*														X								X		
**Impala**	***Aepyceros melampus***	**X**	**X**	**X**	**X**	**X**	**X**	**X**	**X**	**X**	**X**	**X**	**X**	**X**	**X**	**X**	**X**	**X**	**X**	**X**	**X**	**X**	**X**	**X**	**X**
Jackal (Side-striped, Golden, Black-backed)	*Canis adustus*, *C*. *aureus*, *C*. *mesomelas*	X	X		X	X		X		X			X		X	X	X	X		X	X	X	X	X	X
Vervet monkey	*Chlorocebus pygerythrus*										X			X	X						X		X	X	X
African civet	*Civettictis civetta*	X	X	X	X								X			X	X		X		X		X		X
**Wildebeest**	***Connochaetes taurinus***	**X**				**X**	**X**	**X**	**X**	**X**		**X**						**X**	**X**			**X**	**X**	**X**	**X**
**Plains zebra**	***Equus quagga***	**X**	**X**		**X**	**X**	**X**			**X**				**X**	**X**	**X**	**X**	**X**			**X**	**X**	**X**	**X**	**X**
**Thomson's gazelle**	***Eudorcas thomsonii***	**X**				**X**	**X**	**X**	**X**	**X**	**X**	**X**	**X**	**X**				**X**	**X**	**X**	**X**	**X**	**X**	**X**	**X**
Caracal	*Caracal caracal*															X	X								
African wildcat	*Felis lybica*	X	X	X		X	X	X		X	X	X		X					X	X			X	X	
Serval	*Leptailurus serval*				X		X	X	X				X			X					X			X	
Greater bushbaby	*Otolemur crassicaudatus*														X							X	X		
Genet (Small-spotted, Large-spotted)	*Genetta genetta*, *G*. *maculata*		X												X	X	X		X		X	X	X		X
**Maasai giraffe**	***Giraffa camelopardalis***	**X**	**X**	**X**	**X**	**X**	**X**	**X**	**X**			**X**		**X**	**X**	**X**		**X**	**X**	**X**	**X**	**X**	**X**	**X**	**X**
Dwarf Mongoose	*Helogale parvula*										X		X	X	X								X	X	
Mongoose (Egyptian, Slender)	*Herpestes ichneumon*, *Galerella sanguinea*			X	X						X		X		X		X			X	X		X	X	X
**Hyena (Striped, Spotted)**	***Hyaena hyaena*, *Crocuta crocuta***	**X**	**X**	**X**	**X**	**X**	**X**	**X**	**X**	**X**	**X**	**X**	**X**	**X**	**X**	**X**	**X**	**X**	**X**	**X**	**X**	**X**	**X**	**X**	**X**
Porcupine	*Hystrix cristata*	X	X	X	X	X	X		X					X	X	X	X	X	X		X	X	X	X	X
White-tailed mongoose	*Ichneumia albicauda*			X	X				X					X								X	X	X	X
Zorilla	*Ictonyx striatus*			X												X									X
Hare (Cape, Scrub, Spring)	*Lepus capensis*, *L*. *saxatilis*, *Pedetes capensis*	X			X		X		X		X	X	X	X				X	X	X	X	X	X	X	X
**Elephant**	***Loxodonta africana***	**X**	**X**	**X**	**X**	**X**	**X**				**X**			**X**					**X**			**X**	**X**	**X**	**X**
Wild dog	*Lycaon pictus*																					X	X		
**Kirk's dik-dik**	***Madoqua kirkii***	**X**	**X**	**X**	**X**	**X**	**X**	**X**	**X**	**X**	**X**	**X**	**X**	**X**	**X**	**X**	**X**	**X**	**X**	**X**	**X**	**X**	**X**	**X**	**X**
Honey badger	*Mellivora capensis*		X		X	X				X		X										X		X	X
Banded mongoose	*Mungos mungo*		X	X	X																X			X	X
Klipspringer	*Oreotragus oreotragus*		X	X										X	X	X	X								
**Aardvark**	***Orycteropus afer***	**X**	**X**	**X**	**X**	**X**	**X**	**X**	**X**	**X**	**X**	**X**	**X**	**X**	**X**	**X**	**X**	**X**	**X**	**X**	**X**	**X**	**X**	**X**	**X**
Bat-Eared Fox	*Otocyon megalotis*												X				X				X	X	X		X
African lion	*Panthera leo*													X				X	X						
Leopard	*Panthera pardus*	X			X								X	X	X		X	X		X		X		X	X
Olive baboon	*Papio anubis*	X	X	X	X				X					X	X	X	X					X	X	X	X
**Warthog**	***Phacochoerus africanus***	**X**	**X**	**X**	**X**	**X**	**X**							**X**	**X**	**X**	**X**	**X**		**X**	**X**	**X**	**X**	**X**	**X**
**Bushpig**	***Potamochoerus larvatus***	**X**	**X**	**X**	**X**	**X**	**X**	**X**	**X**			**X**		**X**	**X**	**X**	**X**	**X**	**X**	**X**	**X**	**X**	**X**	**X**	**X**
Hyrax (Tree, Bush, Rock)	*Dendrohyrax arboreus*, *Heterohyrax brucei*, *Procavia johnstoni*													X	X	X	X								
Steenbok	*Raphicerus campestris*			X				X								X								X	
Bohor reedbuck	*Redunca redunca bohor*								X															X	
Bush duiker	*Sylvicapra grimmia*	X	X				X									X									
Buffalo	*Syncerus caffer*	X	X		X									X								X			X
**Lesser kudu**	***Tragelaphus imberbis***	**X**	**X**	**X**	**X**	**X**	**X**	**X**	**X**		**X**	**X**		**X**	**X**	**X**	**X**	**X**	**X**	**X**	**X**	**X**	**X**	**X**	**X**
**Eland**	***Tragelaphus oryx***	**X**	**X**	**X**	**X**	**X**	**X**	**X**	**X**	**X**		**X**	**X**	**X**	**X**	**X**	**X**	**X**	**X**	**X**	**X**	**X**	**X**	**X**	**X**
**Bushbuck**	***Tragelaphus scriptus***	**X**	**X**	**X**	**X**	**X**			**X**	**X**			**X**	**X**	**X**	**X**	**X**		**X**			**X**	**X**	**X**	**X**
**Greater kudu**	***Tragelaphus strepsiceros***	**X**	**X**	**X**	**X**	**X**	**X**	**X**	**X**	**X**				**X**	**X**	**X**	**X**	**X**	**X**	**X**	**X**	**X**	**X**	**X**	**X**
*Potential species*																									
Hartebeest	*Alcelaphus buselaphus*																								
Roan antelope	*Hippotragus equinus*																								
Aardwolf	*Proteles cristata*																								
Pangolin	*Smutsia temminckii*																								
*Wildlife species richness*		24	24	22	25	19	19	15	18	13	12	13	15	26	24	24	22	18	19	16	22	27	30	30	30
*Proportion of expected mammal community*		.51	.51	.47	.53	.40	.40	.32	.38	.28	.26	.28	.32	.55	.51	.51	.47	.38	.40	.34	.47	.57	.64	.64	.64

### Data analyses

Completeness of overall mammal species richness in Yaeda Valley was evaluated by comparing confirmed species presence to a list of expected mammal species in the area, which was derived from a recent field guide for mammals in Tanzania [51]. Mammal species richness estimates were calculated for all study year-stratum combinations using the first order Jackknife estimates generated in EstimateS 9.1 [62]. Species accumulation curves as functions of sampling effort (number of walked transects) were graphed using R 3.3.2 [63]. To compare species richness between strata and years, we ran a general linear model (glm) on estimated species richness at highest common sampling effort (11 transects). Since the response variable was normally distributed (Shapiro-Wilk test: W = 0.962, p = 0.484), we used the Gaussian error distribution for the glm. We first fitted the most complex model (interaction of stratum x year) to explain differences in species richness, then generated all subsets of this model using the *dredge* function of the *MUMIn* package and finally selected the most supported model based on the second order AIC-score [64].

Mammal densities based on direct sightings and relative densities based on signs in each stratum and year were estimated using DISTANCE 6.0 [65]. For each species, four different global detection models (uniform, half-normal, hazard-rate, and negative-exponential) were fitted to species-specific data using conventional distance sampling. Chi-squared goodness of fit values of the detection functions were frequently significant, which implied poor fit of detection functions [66,67]. Because half-normal detection functions had a general acceptable visual fit, we selected half-normal detection functions for all species. Since relatively few direct mammal sightings were recorded, and distance sampling methodology requires a relatively high number of observations (>60 observations) to reliably estimate detection functions [67], detection functions were based on the entire dataset, and densities were only estimated for the five most frequently directly encountered species, three of which were livestock. Livestock and Thomson’s gazelle (*Eudorcas thomsonii*) exceeded the recommended observation threshold, and Kirk’s dik-dik (*Madoqua kirkii*) was included despite only 40 direct sightings. Fourteen species were selected for sign density estimations, all of which exceeded the threshold of 60 observations. Stratum- and year-specific densities were estimated using the post-stratification option in the distance software and were inferred based on mean cluster sizes in each stratum. We assessed temporal density differences in each stratum between 2015 and 2018, testing the null hypothesis that density did not change over time, using a generalized linear model with log transformed error distribution [68]. If a species was not detected in a given year-stratum combination, we converted the estimated zero density to 0.01 to allow log transformation. Given that the test power for 4 years of monitoring is likely weak, we did not strictly focus on p-value testing but instead graphically compared yearly regression coefficients (i.e. yearly population growth rates) across strata. For this comparison, we excluded species with only one density estimate in time that exceeded zero signs or individuals per km^2^. This was done to avoid assigning strong weight to possible outliers in population growth rates that may have been caused by observations of species that do not regularly occupy a given stratum.

## Results

### Mammal species richness

Over the course of four years, we detected a total of 9007 independent observations of signs and 346 direct sightings of wildlife species. We observed signs from 43 wildlife species (groups), with direct sightings of 27 species (S1 Data). Compared to 47 expected terrestrial mammal species (groups) in the region, our survey confirmed that a near complete (91.5%) mammal species assemblage occurred in the area.

**Fig 2:**
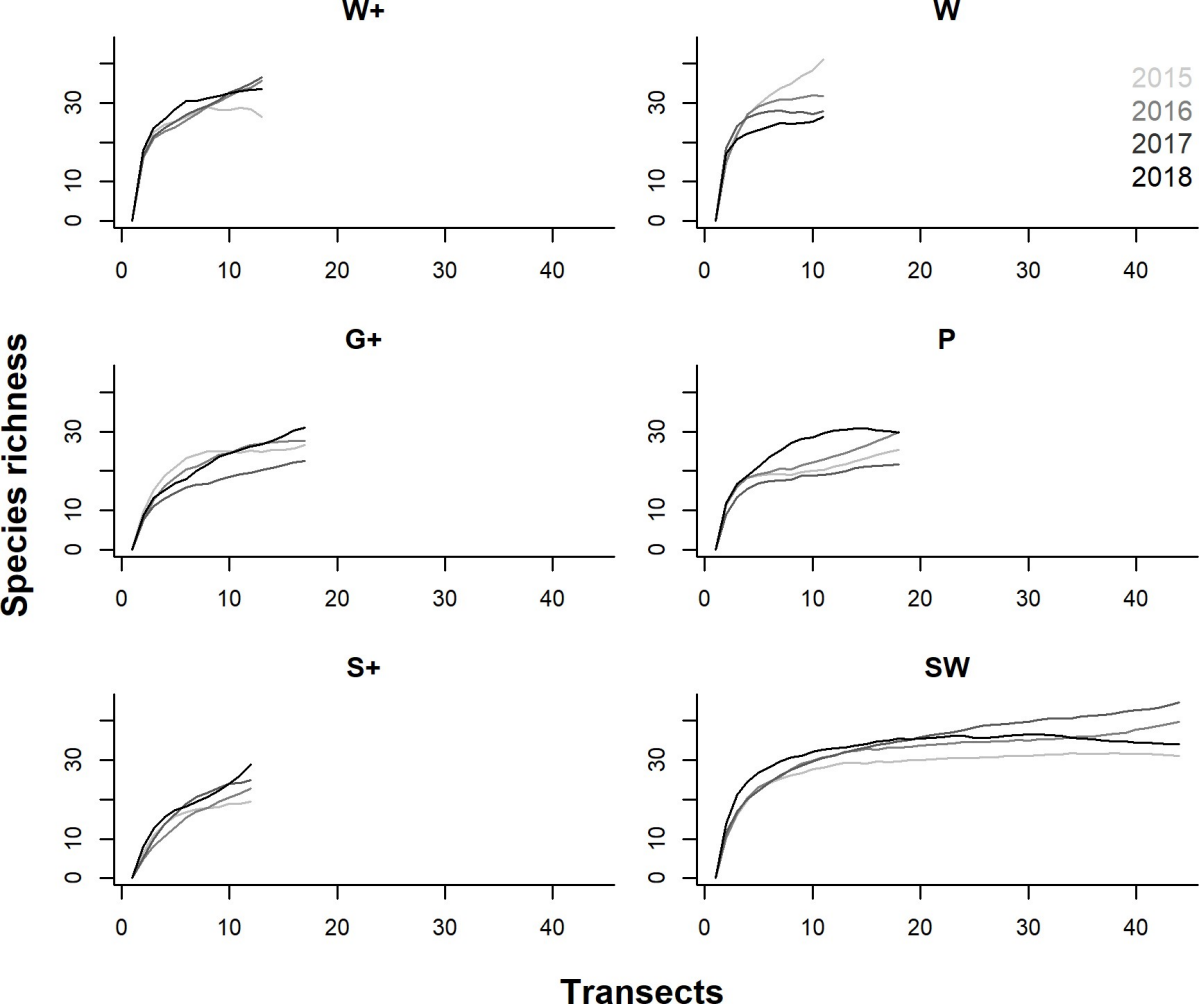
First-order Jackknife estimates of mammal species richness in Yaeda Valley. Species richness estimates were plotted against sampling effort in the three REDD+ and the three control strata in 2015, 2016, 2017 and 2018.

Stratum-specific species rarefaction curves were mostly asymptotic (Fig 2), suggesting that sampling efforts in most strata were sufficient. Estimated mammal species richness was highest in W+, W, and SW compared to other strata in all survey years, and stratum-specific species richness estimates exhibited variability across years (Fig 2).

Model selection suggested that the most supported model to explain variation in species richness (at smallest common sampling effort of 11 transects) contained the factor “stratum” only (Table 2A). This analysis confirmed that mammal species richness was highest in the three woodland strata (W+, W, SW), and was significantly lower in the plains habitats (G+, P) and in the settlement area (S+) (Table 2B). Incorporating survey year in the model was not supported based on information-theoretic model weights, suggesting that species richness did not differ substantially across survey years (Table 2A). Although not statistically significant, mammal species richness appears to have declined linearly over time in stratum W (Fig 2).

**Table 2:** (a) Model selection table and (b) regression coefficients of the most supported general linear model to explain spatio-temporal variation in mammal species richness in Yaeda Valley. Species richness was estimated in six strata [three REDD+ strata (W+, G+, and S+) three control land-use strata (W, P, and SW) in 2015, 2016, 2017, and 2018. The response variable was estimated for a sampling effort of n = 11 transects in each stratum.

(a)	Intercept	Stratum	Year	Stratum*Year	df	logLik	AICc	delta AICc	weight
	30.54	+			7	-63.438	147.9	0	0.831
	-757.2	+	0.3907		8	-63.238	152.1	4.2	0.102
	27.25				2	-74.433	153.4	5.56	0.051
	-760.5		0.3907		3	-74.354	155.9	8.03	0.015
	-2631	+	1.32	+	13	-49.447	161.3	13.42	0.001
(b)		Estimate	Std. error	P-value					
	Intercept (SW)	30.535	1.964	≤0.001					
	Stratum P	-7.618	2.778	0.013					
	Stratum W	1.247	2.778	0.659					
	Stratum W+	1.292	2.778	0.647					
	Stratum G+	-6.818	2.778	0.025					
	Stratum S+	-7.825	2.778	0.011					

### Mammal population and sign densities

Stratum- and year-specific population densities were estimated for livestock species and two of the most frequently encountered wildlife species (S1 Data) using selected detection functions summarized in S2 Data and displayed S1 Fig Sign densities of wildlife species were estimated using half-normal detection functions (S2 Data) which are displayed in the S2 Fig Frequent significant signals of chi-square goodness of fit tests (direct sightings: 2/5 species; signs: 15/15 species) suggested relatively poor statistical fit of the selected detection functions (S2 Data). However, apart from a spike of observations near the transect lines (which likely caused significant goodness of fit results), visual fit of the fitted detection function appeared to match the observed frequencies of observations (S1 and S2 Figs).

**Fig 3:**
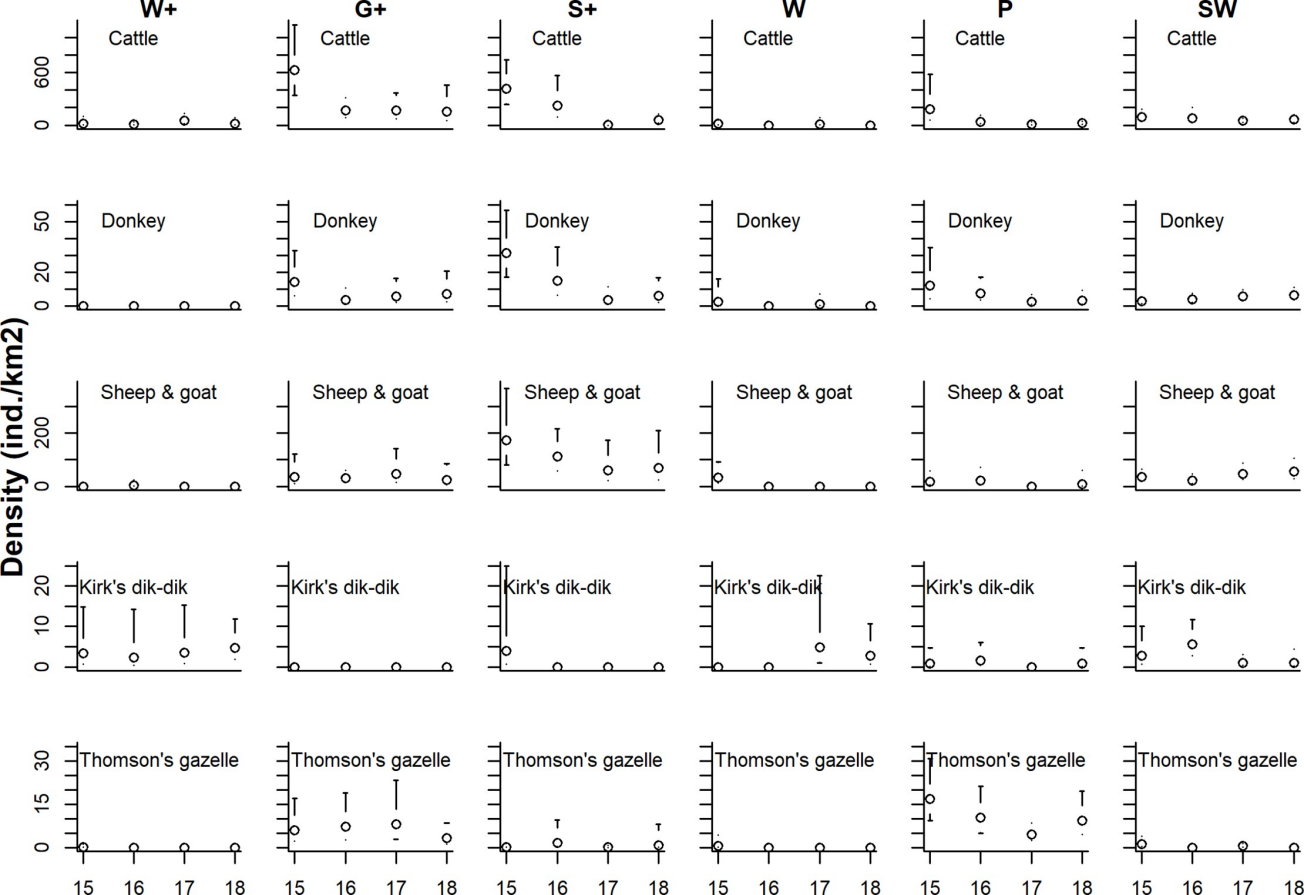
Population density estimates of livestock species, Thomson’s gazelle, and Kirk’s dik- dik in Yaeda Valley, Tanzania. Density estimates were stratified by stratum [three REDD+ strata (W+, G+, and S+), three control land-use strata (W, P, and SW)], and year. Error bars indicate 95% confidence intervals.

Densities of directly sighted mammal species were highest for livestock species, particularly for cattle in G+ and S+, and sheep and goats in S+ (Fig 3). Densities of Kirk’s dik-dik were particularly high in W+ and Thomson’s gazelle mainly occurred at high densities in the grassland strata (G+, P).

**Fig 4:**
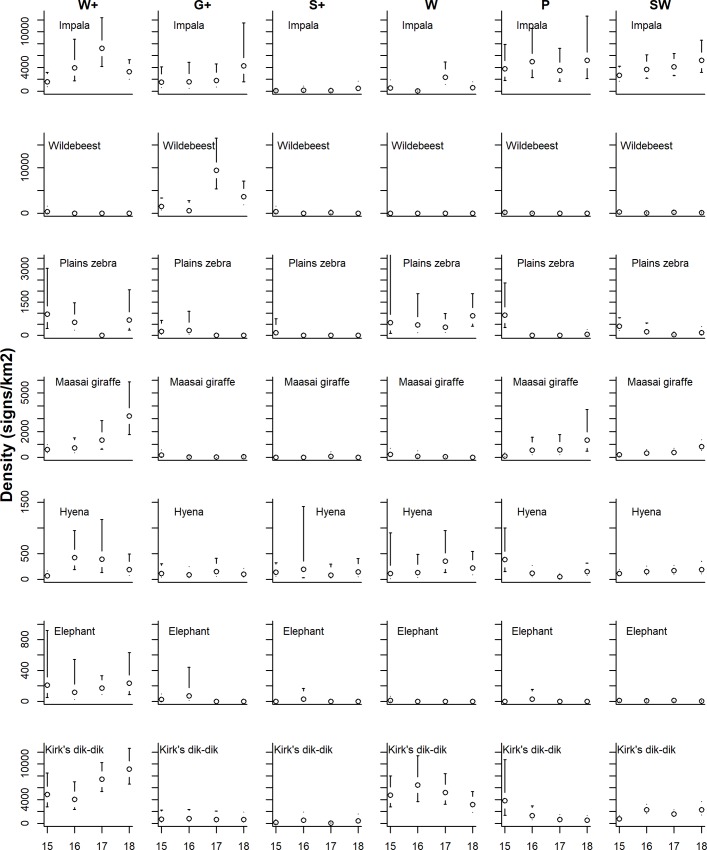
Sign density estimates of impala, wildebeest, Maasai giraffe, hyena, elephant and Kirk’s dik-dik in Yaeda Valley, Tanzania. Density estimates were stratified by stratum [three REDD+ strata (W+, G+, and S+), three control land-use strata (W, P, and SW)], and year. Error bars indicate 95% confidence intervals.

**Fig 5:**
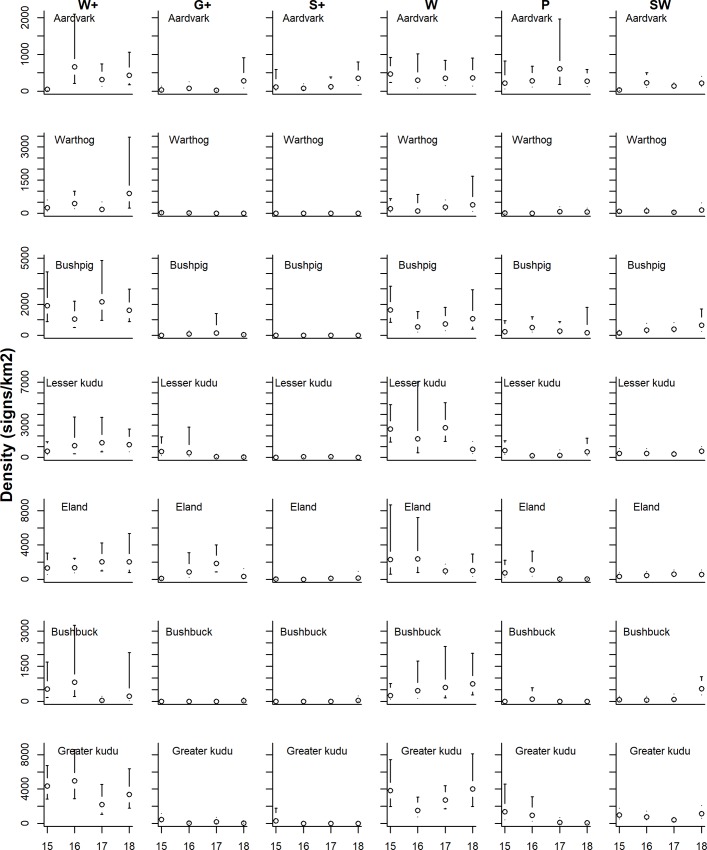
Sign density estimates of aardvark, warthog, bushpig, lesser kudu, eland, bushbuck, and greater kudu in Yaeda Valley, Tanzania. Density estimates were stratified by stratum [three REDD+ strata (W+, G+, and S+), and three control land-use strata (W, P, and SW)] and year. Error bars indicate 95% confidence intervals.

Estimated sign densities indicated that several species occurred at relatively high densities across the landscape. For example impala sign densities were relatively high in most strata except for S+ (Fig 4; S3 Data), and hyena signs were relatively high in all strata (Fig 4). Several species (e.g. Plains zebra, greater and lesser kudu, eland) occurred at relatively high densities in multiple, but not all, strata (Fig 4, Fig 5) whereas other species had rather restricted distributions. For example, wildebeest solely occurred in G+, elephants mainly in W+ and SW, Maasai giraffe primarily occurred in W+ and P (Fig 4), and bushbuck sign densities were high in W+ and W but absent or very low in other strata (Fig 4, Fig 5).

### Wildlife population trends

**Fig 6:**
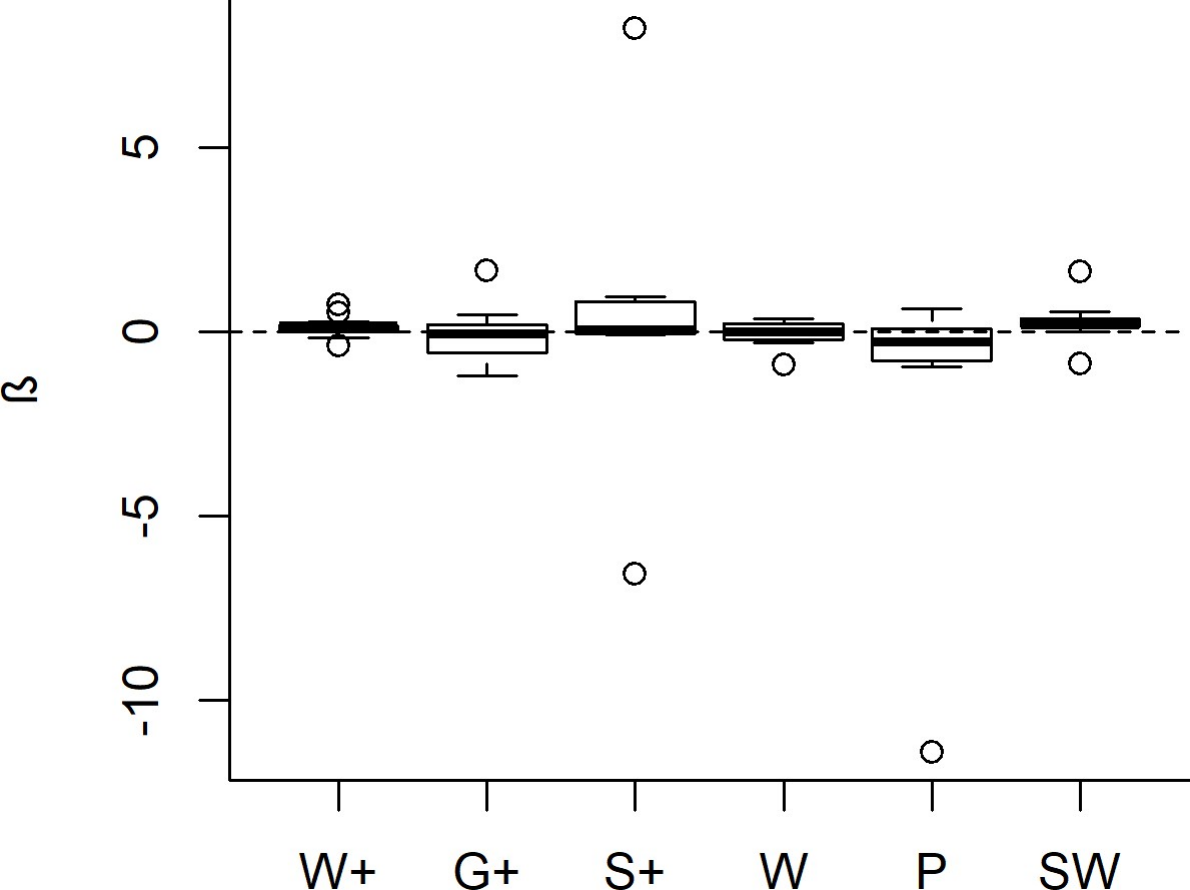
Boxplot showing regression coefficients of annual wildlife population growth rates (β) in Yaeda Valley, Tanzania. Species-specific regression coefficients of β were estimated using a generalized linear model with log-link and were estimated independently for each land-use stratum [three REDD+ strata (W+, G+, and S+) and three control land-use strata (W, P, and SW)]. The dashed line indicates zero (i.e. no population change).

Population densities of observed livestock and wildlife species as well as sign densities of the more commonly encountered wildlife species exhibited relatively strong temporal patterns over the last four years (Figs 3–6). In particular, declines in livestock densities in the G+ and S+ strata are noteworthy (Fig 3; S4 Data). Estimates for yearly population growth rates were variable across species and strata (Fig 6, S4 Data). The variation in growth rates was, however not significantly associated with land-use stratum (Kruskal-Wallis Anova: Χ^2^ = 8.77; df = 5; p = 0.12). Among species-stratum combinations that demonstrated strong temporal patterns (using p ≤ 0.1 as criteria), most of the wildlife population growth rates (9/12) were positive and occurred both in the core REDD+ stratum (W+: n = 2) as well as in non-REDD+ strata (W: n = 1; P: n = 1; SW: n = 5). Particularly interesting is the population trajectory of Maasai giraffe which apparently increased in W+, P, SW but declined in W (S4 Data).

### Discussion

Our ground-based monitoring results reveal that Yaeda Valley, an area partially protected via a REDD+ project, supports a near complete mammal community including charismatic species of conservation concern such as African elephants [69], African lions [70,71], cheetahs [72] and wild dogs [73]. Results of the stratified sampling approach confirm relatively strong habitat-species associations and suggest that wildlife populations in our study area are mostly stable or increasing. Although the REDD+ land-use plan may have contributed to this positive conservation outcome, rates of change in wildlife populations were not strictly associated with REDD+ land-use strata as they occurred in other land-use strata as well.

### Patterns of mammal species richness

Habitat protection via REDD+ mechanisms likely plays a role in sustaining a near complete savanna mammal community in Yaeda Valley. This is demonstrated particularly by the highest species richness estimates in the W+, a near pristine (basically no land conversion) dryland savanna habitat where the main land use is foraging (hunting, honey collection, fruit and tuber gathering) by Hadza people [56,74]. Other strata (particularly S+; G+; P) had substantially higher human influences, indicated by higher livestock densities (Fig 4) and consequently -similar to other studies along conservation gradients in northern Tanzania—lower mammal species richness [75,76]. Interestingly, and in line with mammal diversity surveys conducted in Malaysia [77], our study suggests that wooded habitats (W+, W, SW) yield the highest conservation value in terms of mammal species richness. However, this finding contrasts a recent study conducted in Botswana, which found grasslands supporting the greatest large mammal species richness [78]. Possibly, the discrepancy between these studies may be explained by presumably higher overall livestock and human presence in grassland habitats in our study and a rather degraded state of the grasslands during the time of our surveys compared to the (possibly more protected) study sites in Botswana [78].

Beyond effective habitat conservation in parts of our study area, several other factors may promote high mammal species richness in Yaeda Valley. The area is relatively large in size (~2500 km^2^), and contains multiple habitat types which likely promotes large mammal persistence [79–81]. In line with this argument, the area is structurally connected to Maswa Game Reserve and Ngorongoro Conservation Area [20] and thus likely facilitates the movement of wide ranging animals such as wild dogs, cheetahs and elephants in and out of the area which may additionally ensure the persistence of even rare species [17].

Certain mammal species, which were not detected (pangolin and aardwolf) may actually be present in the study area but probably occur at very low densities and are thus very difficult to detect with sample-based field methods [82]. Historically, the area also contained black rhinoceros, roan antelope and hartebeest [56]. While black rhinoceros most likely went locally extinct due to illegal hunting (and indeed rhinoceros do not occur outside selected fully protected areas in Tanzania and were thus not listed as potentially occurring in the area), one can only speculate as to why roan antelope and hartebeest apparently went locally extinct in this area.

### Patterns of wildlife densities

**Fig 7:**
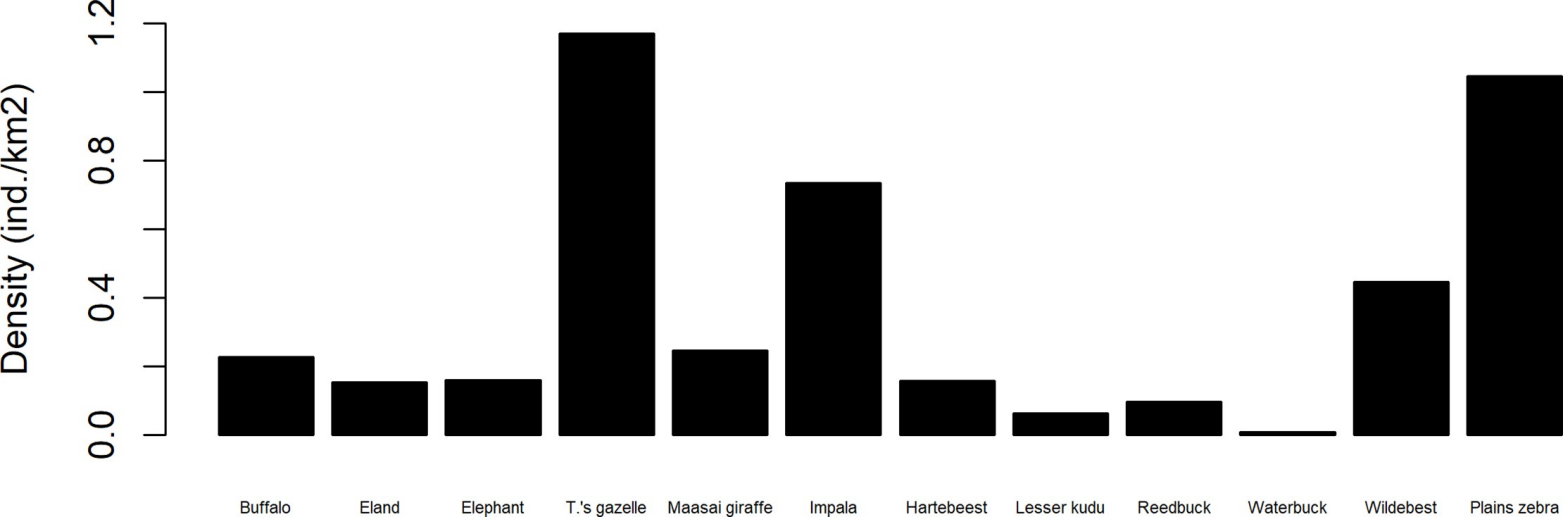
Mean wildlife population densities in Yaeda Valley, Tanzania estimated from three systematic reconnaissance flight surveys conducted in 1977, 1978 and 1980. Data on population densities have been obtained from [85].

Despite intensive field efforts each year, most of the mammal species were either rarely seen directly (S1 Data) or only indirectly detected via spoor, dung, or feeding signs. This overall pattern may be explained by two mutually non-exclusive hypotheses: actual low wildlife density and/or evasive animal behavior towards humans. Indeed, wildlife may be expected to occur at low densities in this semi-arid area given the close relationships between rainfall, primary productivity, and herbivore and carnivore density [83,84]. However, anecdotal information suggest that wildebeest, zebra and elephant densities were previously much greater than nowadays [56]. Unfortunately, directly comparing density estimates derived from aerial surveys conducted from 1978–1980 in the general area of Yaeda Valley (Fig 7) is not feasible due to methodological and coverage differences, yet the data highlight the historical dimensions of wildlife populations in the area.

The survey set-up did not allow inferring actual animal densities from densities of animal signs [86,87] due to the logistical problems associated with estimating species-specific sign (tracks, dung, feeding signs) production and decomposition rates [88–90] for an entire mammal community. However, based on naive sign densities, several ungulate species appear to occur at relatively high densities, particularly in the W+ and W (Figs 4 and 5), but were rarely or never seen directly (S1 Data). This pattern points to the idea that many animal species are shy and avoid being detected by humans, especially in human-dominated areas. Provided that animals in the area have been hunted by Hadza people with bows and arrows for millennia [59,74], behavioral adaptations in response to this form of persecution have likely evolved [91] and may explain low direct encounter rates of wildlife.

Albeit monitoring indirect signs has substantial advantages in this setting, the monitoring techniques could be improved. For example, in the future (and already implemented in the 2018 survey), randomization of the transect line (e.g. by dropping a walking stick on the transect line upon encountering a wildlife sign) could be used to avoid the observed spike near the transect line and to increase the statistical fit of detection functions (S1 and S2 Figs [92]).

### Wildlife population trends

Analyses of repeated surveys suggest that livestock densities varied considerably across years. In particular, cattle densities in the plains habitats (G+, S+) were substantially higher in 2015 compared to 2017. Most likely, this can be explained by invasions of nomadic pastoralists from other regions to the Yaeda area for grazing during 2015. Government interventions likely led to a reduction in this external influx of livestock. Clearly, such added livestock densities may increase grazing pressure on resident livestock populations and on grazing wildlife species [93], and create tension between nomadic pastoralists, residents of the area, and overall conservation goals. On the whole, our surveys confirm that land-use policies are largely implemented, albeit livestock grazing is occasionally observed in W+, where land-use plans do not permit this practice (Fig 3).

Yearly density fluctuations in wildlife (sign) densities were observed in multiple wildlife species (Figs 3–6, S4 Data). These shifts may have been caused by temporal distribution shifts of wildlife species in response to variable resource availability and/or pressures exerted by livestock and humans. This temporal variability in wildlife densities may further suggest that spill-over effects from one area to another are likely. Thus, when using mammals (which can move over large distances) as indicators of conservation effectiveness, large-scale and repeated wildlife monitoring surveys are required for making meaningful assessments [94].

Eventually, long-term monitoring may indicate which species benefit (species with increasing population trends) and which may not be sufficiently protected by REDD+ mechanisms [33]. Among apparent “winners” of REDD+ conservation schemes, are Kirk’s dik dik and Maasai giraffe which appear to thrive particularly well in the core REDD+ area W+. Both species are strict browsers [95] and actively conserving woody plants is likely to benefit these species [53]. However, giraffe populations appear to increase in other strata as well (P and SW; Fig 4) which may point to the idea that additional causal factors may underlie this apparent population increase. Beyond habitat protection, the REDD+ project employs village game scouts, which patrol the area on foot and ensure adherence to land-use policies (i.e. enforcing livestock restrictions, illegal hunting—which in this area is all hunting carried out by non-Hadza ethnicities). It is possible that these anti-poaching efforts have contributed to the overall conservation of the area and have substantially contributed to stable or increased population densities of most wildlife species as well. Our study did not identify apparent “loser species” of REDD+ conservation schemes but by design, the REDD+ scheme does not directly benefit species that rely primarily on grass (e.g. warthogs, wildebeest, Plains zebra), yet these species may benefit indirectly from the REDD+ enforced land-use plans which include designated grazing areas (where settlements are not allowed), moderate livestock densities, and areas where livestock are excluded.

REDD+ and other forest-oriented conservation schemes are now mainstream, global conservation approaches and yet they frequently lack wildlife monitoring schemes [33,77,96]. Although four years of wildlife monitoring may not be sufficient to detect major population changes in large mammal populations, given the often slow, and often time-lagged responses of large mammal species to changes in the environment and conservation policies [53], our results imply that REDD+ based land-use plans can—at least—contribute to sustaining species rich wildlife assemblages in East African rangelands.

### Conservation implications

Beyond being of intrinsic value, large mammals provide a multitude of quantifiable ecosystem services, including protein supply for indigenous hunting and gatherer societies, and are thus of critical socio-economic importance [74]. In addition, mammal species are crucial for nutrient cycling and seed dispersal up to the point that defaunation (i.e. the loss of large mammals) has the potential of reducing landscape levels of carbon stocks [97]. Henceforth, investment in conserving intact animal communities directly benefits carbon storage and thus supports the principal goal of REDD+. The few existing wildlife assessments conducted in REDD+ areas [33,96] suggest that wildlife conservation in REDD+ areas is feasible, but may require additional efforts to explicitly address threats to wildlife populations beyond habitat loss such as competition with livestock during times of resource scarcity, and illegal and unsustainable hunting. In Yaeda Valley, village game scouts hired under the REDD+ scheme also enforce anti-poaching laws when patrolling the area on foot and received additional law-enforcement training in 2017. However, the effectiveness of these patrols is likely limited due to the lack of logistical support such as the lack of modern transportation means. Hence, a more formal and specific integration of biodiversity conservation goals under the REDD+ scheme, including specific monitoring programs and incentive based payments for achieving clearly defined wildlife and overall biodiversity conservation goals may substantially improve the ability of REDD+ projects to directly address threats to biodiversity conservation beyond habitat loss [98,99].

## Supporting information

S1 Data Number of independent wildlife sign detections (Signs) and number of wildlife and livestock sightings (Sightings) in Yaeda Valley from 2015–2018. (DOCX)

S2 Data Summary of half-normal detection functions to estimate animal densities and animal sign densities in Yaeda Valley. Reported parameters for each model include number of encounters within truncation distance (n), global detection probability (P_a_) and associated 95% confidence intervals (P_a_-lower—P_a_-upper), estimated strip width, and corresponding chi-squared goodness of fit (GOF)–p-value.
(DOCX)

S3 Data Estimated stratum- and year-specific animal and animal sign densities in Yaeda Valley, Tanzania. Densities based on sightings were indicated by “S” following the species name, other densities indicate sign densities. Columns with the letter “L” following the species name indicate the lower 95% confidence interval for the density estimate, columns with the letter “U” following the species name, indicate the upper 95%-confidence intervals of the density estimates.
(DOCX)

S4 Data Regression coefficient estimates (β) and associated p-values for the effect of year on population density estimates of livestock and wildlife species in Yaeda Valley, Tanzania. Population growth rates were estimated from 2015 to 2018 for all six strata [three REDD+ strata (W+, G+, and S+) three control land-use strata (W, P, and SW)] using generalized linear models with log-link. Empty cells denote that the species were not detected in this stratum. Regression coefficients for species highlighted in grey were not utilized for comparisons across land-use strata (main manuscript Fig 6) because those species were either livestock species, or had only one estimate in time that exceeded 0 signs or individuals per km^2^. For Kirk’s dik-dik we used the sign density, because signs were observed in all strata.
(DOCX)

S1 Fig Detection functions of direct mammal sightings. Histograms (blue bars) represent sighting frequency and the red line is the fitted detection function.
(TIFF)

S2 Fig Detection functions of mammal signs. Histograms (blue bars) represent sighting frequency and the red line is the fitted detection function.
(TIFF)
