# Supplementary material for: Land use, REDD+ and the status of wildlife populations in Yaeda Valley, northern Tanzania
Source: PLoS One. 2019 Apr 4;14(4):e0214823. doi: 10.1371/journal.pone.0214823 (PMC6448838; doi:10.1371/journal.pone.0214823)
Supplement: S1 Data — (DOCX) [file pone.0214823.s001.docx]

**S1 Data. Number of independent wildlife sign detections (Signs) and number of wildlife and livestock sightings (Sightings) in Yaeda Valley from 2015-2018.**

|  |  | **Signs** | | | |  | **Sightings** | | | |
| --- | --- | --- | --- | --- | --- | --- | --- | --- | --- | --- |
| **Common name** | **Scientific name** | **2015** | **2016** | **2017** | **2018** |  | **2015** | **2016** | **2017** | **2018** |
| *Livestock* |  |  |  |  |  |  |  |  |  |  |
| Cattle | *Bos taurus* |  |  |  |  |  | 153 | 97 | 96 | 93 |
| Domestic dog | *Canis lupus familiaris* |  |  |  |  |  | 22 | 13 | 23 | 18 |
| Donkey | *Equus africanus asinus* |  |  |  |  |  | 78 | 53 | 49 | 51 |
| Domestic cat | *Felis catus* |  |  |  |  |  |  |  | 1 | 1 |
| Sheep & Goat | *Ovis aries & Capra aegagrus hircus* |  |  |  |  |  | 77 | 69 | 57 | 61 |
|  |  |  |  |  |  |  |  |  |  |  |
| *Wildlife* |  |  |  |  |  |  |  |  |  |  |
| Cheetah | *Acinonyx jubatus* |  | 2 |  |  |  |  |  |  |  |
| Impala | *Aepyceros melampus* | 334 | 349 | 521 | 494 |  | 3 | 3 | 7 | 1 |
| Jackal (Side-striped, Golden, Black-backed) | *Canis adustus, C. aureus, C. mesomelas* | 11 | 7 | 26 | 42 |  | 1 | 1 | 1 |  |
| Vervet monkey | *Cercopithecus aethiops* | 2 | 3 | 1 | 2 |  |  | 3 | 2 | 2 |
| African civet | *Civettictis civetta* | 2 | 12 | 4 | 9 |  |  |  |  |  |
| Common wildebeest | *Connochaetes taurinus* | 75 | 22 | 188 | 79 |  | 7 | 1 | 2 | 3 |
| Plain's zebra | *Equus burchelli* | 92 | 39 | 9 | 44 |  | 1 |  |  |  |
| Caracal | *Felis caracal* |  |  | 2 |  |  |  |  |  | 1 |
| African wildcat | *Felis lybica* | 7 | 15 | 10 |  |  |  |  | 2 |  |
| Serval | *Felis serval* |  | 3 | 3 | 7 |  |  |  | 1 |  |
| Greater bushbaby | *Galago crassicaudatus* |  | 7 |  |  |  | 1 |  |  |  |
| Thomson's gazelle | *Eudorcas thomsonii* | 269 | 222 | 311 | 270 |  | 74 | 48 | 44 | 45 |
| Genet (Small-spotted, Large-spotted) | *Genetta genetta, G. maculata* | 2 | 6 | 2 | 6 |  |  | 1 |  |  |
| Maasai giraffe | *Giraffa camelopardalis* | 82 | 103 | 158 | 269 |  |  | 3 |  | 5 |
| Dwarf mongoose | *Helogale parvula* |  | 2 |  | 1 |  | 1 | 1 | 1 |  |
| Mongoose (Egyptian, Slender) | *Herpestes ichneumon, H. sanguineus* |  | 5 | 4 | 19 |  |  | 1 | 1 |  |
| Hyena (Striped, Spotted) | *Hyaena hyaena, Crocuta crocuta* | 59 | 69 | 86 | 98 |  |  |  |  |  |
| Porcupine | *Hystrix cristata* | 18 | 31 | 16 | 28 |  |  |  |  |  |
| White-tailed mongoose | *Ichneumia albicauda* | 1 | 1 | 2 | 9 |  | 1 |  |  |  |
| Zorilla | *Ictonyx striatus* |  |  | 8 | 1 |  |  |  |  |  |
| Hare (Cape, Scrub, Spring) | *Lepus capensis, L. saxatilis, Pedetes capensis* | 2 | 5 |  | 82 |  |  | 1 | 4 | 11 |
| Elephant | *Loxodonata africana* | 31 | 19 | 19 | 15 |  |  |  |  |  |
| Wild dog | *Lycaon pictus* | 1 | 1 |  |  |  |  |  |  |  |
| Kirk's dik-dik | *Madoqua krikii* | 271 | 369 | 347 | 344 |  | 9 | 16 | 7 | 8 |
| Honey badger | *Mellivora capensis* | 6 | 1 | 2 | 3 |  |  |  |  |  |
| Banded mongoose | *Mungus mungo* |  | 2 | 4 | 2 |  |  |  |  | 1 |
| Klipspringer | *Oreotragus oreotragus* | 12 | 2 | 16 | 2 |  |  |  |  |  |
| Aardvark | *Orycteropus afer* | 65 | 112 | 120 | 156 |  |  |  |  |  |
| Bat-eared fox | *Otocyon megalotis* | 1 | 3 |  | 6 |  |  |  |  |  |
| African lion | *Panthera leo* | 2 | 1 |  |  |  |  |  |  |  |
| Leopard | *Panthera pardus* | 5 | 1 | 2 | 9 |  |  |  |  |  |
| Olive baboon | *Papio anubis* | 22 | 13 | 26 | 25 |  | 2 |  |  |  |
| Warthog | *Phacochoerus africanus* | 35 | 30 | 30 | 30 |  |  | 1 |  |  |
| Bushpig | *Potamochoerus porcus* | 66 | 63 | 87 | 94 |  | 3 |  |  |  |
| Hyrax (Bush, Rock, Tree) | *Heterohyrax brucei, Procavia johnstoni, Dendrohyrax arboreus* | 2 | 1 | 11 | 1 |  | 1 | 1 |  |  |
| Steenbok | *Raphicerus campestris* |  |  | 33 |  |  |  |  |  |  |
| Bohor's reedbuck | *Redunca redunca bohor* |  |  | 2 |  |  |  |  |  | 1 |
| Bush duiker | *Sylvicapra grimmia* | 2 | 6 | 5 |  |  |  |  |  |  |
| Buffalo | *Syncerus caffer* | 19 | 1 |  | 19 |  |  |  |  |  |
| Lesser kudu | *Tragelaphus imberbis* | 115 | 71 | 110 | 93 |  |  | 2 | 1 |  |
| Eland | *Tragelaphus oryx* | 117 | 157 | 178 | 142 |  |  |  |  | 1 |
| Bushbuck | *Tragelaphus scriptus* | 23 | 22 | 18 | 68 |  | 2 |  |  |  |
| Greater kudu | *Tragelaphus strepsiceros* | 205 | 169 | 105 | 169 |  |  |  | 2 | 3 |
