# Supplementary material for: Land use, REDD+ and the status of wildlife populations in Yaeda Valley, northern Tanzania
Source: PLoS One. 2019 Apr 4;14(4):e0214823. doi: 10.1371/journal.pone.0214823 (PMC6448838; doi:10.1371/journal.pone.0214823)
Supplement: S2 Data — Reported parameters for each model include number of encounters within truncation distance (n), global detection probability (Pa) and associated 95% confidence intervals (Pa-lower—Pa-upper), estimated strip width, and corresponding chi-squared goodness of fit (GOF)–p-value. (DOCX) [file pone.0214823.s002.docx]

**S2 Data.** **Summary of half-normal detection functions to estimate animal densities and animal sign densities in Yaeda Valley.** Reported parameters for each model include number of encounters within truncation distance (n), global detection probability (P_a_) and associated 95% confidence intervals (P_a_-lower - P_a_-upper), estimated strip width, and corresponding chi-squared goodness of fit (GOF) – p-value.

| **Species** | **n** | **P_a_** | **P_a_ -lower** | **P_a_ -upper** | **Estimated strip width (m)** | **Main function** | **P-value** |
| --- | --- | --- | --- | --- | --- | --- | --- |
| Cattle - sightings | 399 | 0.41 | 0.37 | 0.46 | 110.29 | Half Normal | 0.0004 |
| Donkey - sightings | 211 | 0.44 | 0.38 | 0.52 | 105.58 | Half Normal | 0.1908 |
| Sheep & goat - sightings | 238 | 0.46 | 0.39 | 0.54 | 83.76 | Half Normal | 0.0613 |
| Thomson's gazelle - sightings | 192 | 0.63 | 0.55 | 0.72 | 217.57 | Half Normal | 0.1306 |
| Dik-dik - sightings | 39 | 0.31 | 0.21 | 0.46 | 17.5 | Half Normal | 0.0047 |
|  |  |  |  |  |  |  |  |
| Impala - signs | 1529 | 0.27 | 0.26 | 0.29 | 0.88 | Half Normal | <0.0001 |
| Wildebeest - signs | 327 | 0.25 | 0.22 | 0.28 | 1.13 | Half Normal | <0.0001 |
| Plain's zebra - signs | 184 | 0.2 | 0.78 | 0.23 | 0.61 | Half Normal | <0.0001 |
| Thomson's gazelle - signs | 243 | 0.39 | 0.34 | 0.45 | 1.14 | Half Normal | <0.0001 |
| Maasai giraffe - signs | 553 | 0.26 | 0.23 | 0.28 | 1.02 | Half Normal | <0.0001 |
| Hyena - signs | 281 | 0.39 | 0.35 | 0.44 | 1.19 | Half Normal | <0.0001 |
| Elephant - signs | 76 | 0.31 | 0.26 | 0.38 | 2.58 | Half Normal | <0.0001 |
| Kirk's dik-dik - signs | 1196 | 0.23 | 0.22 | 0.25 | 0.72 | Half Normal | <0.0001 |
| Aardvark - signs | 409 | 0.35 | 0.32 | 0.39 | 2.43 | Half Normal | 0.0017 |
| Warthog - signs | 112 | 0.33 | 0.28 | 0.38 | 1.22 | Half Normal | <0.0001 |
| Bushpig - signs | 278 | 0.33 | 0.3 | 0.37 | 0.61 | Half Normal | <0.0001 |
| Lesser kudu - signs | 350 | 0.27 | 0.24 | 0.31 | 0.57 | Half Normal | <0.0001 |
| Eland - signs | 535 | 0.26 | 0.23 | 0.28 | 0.74 | Half Normal | <0.0001 |
| Bushbuck - signs | 118 | 0.44 | 0.36 | 0.53 | 0.56 | Half Normal | <0.0001 |
| Greater kudu - signs | 592 | 0.24 | 0.22 | 0.26 | 0.48 | Half Normal | <0.0001 |
