# Supplementary material for: Land use, REDD+ and the status of wildlife populations in Yaeda Valley, northern Tanzania
Source: PLoS One. 2019 Apr 4;14(4):e0214823. doi: 10.1371/journal.pone.0214823 (PMC6448838; doi:10.1371/journal.pone.0214823)
Supplement: S3 Data — Densities based on sightings were indicated by “S” following the species name, other densities indicate sign densities. Columns with the letter “L” following the species name indicate the lower 95% confidence interval for the density estimate, columns with the letter “U” following the species name, indicate the upper 95%-confidence intervals of the density estimates. (DOCX) [file pone.0214823.s003.docx]

**S3 Data.** **Estimated stratum- and year-specific animal and animal sign densities in Yaeda Valley, Tanzania.** Densities based on sightings were indicated by “S” following the species name, other densities indicate sign densities. Columns with the letter “L” following the species name indicate the lower 95% confidence interval for the density estimate, columns with the letter “U” following the species name, indicate the upper 95%-confidence intervals of the density estimates.

| Stratum | Year | Cattle S | Cattle SL | Cattle SU | Sheep&goat S | Sheep&goat SL | Sheep&goat SU | Donkey S | Donkey SL | Donkey SU |
| --- | --- | --- | --- | --- | --- | --- | --- | --- | --- | --- |
| W+ | 2015 | 16.25 | 2.65 | 99.86 | 0.00 |  |  | 0.00 |  |  |
| W+ | 2016 | 8.41 | 1.98 | 35.64 | 4.05 | 0.66 | 24.76 | 0.00 |  |  |
| W+ | 2017 | 51.18 | 19.07 | 137.36 | 0.00 |  |  | 0.00 |  |  |
| W+ | 2018 | 14.40 | 2.36 | 88.03 | 0.00 |  |  | 0.00 |  |  |
| G+ | 2015 | 625.45 | 343.18 | 1139.90 | 35.05 | 10.07 | 121.91 | 13.98 | 5.95 | 32.86 |
| G+ | 2016 | 166.19 | 88.13 | 313.38 | 29.77 | 14.81 | 59.86 | 3.60 | 1.24 | 10.43 |
| G+ | 2017 | 165.27 | 74.50 | 366.62 | 46.12 | 15.10 | 140.89 | 5.44 | 1.83 | 16.15 |
| G+ | 2018 | 154.00 | 52.03 | 455.86 | 23.01 | 6.21 | 85.27 | 6.95 | 2.33 | 20.76 |
| S+ | 2015 | 418.14 | 235.67 | 741.88 | 172.90 | 80.96 | 369.26 | 31.23 | 17.15 | 56.87 |
| S+ | 2016 | 225.94 | 90.59 | 563.51 | 111.94 | 57.91 | 216.38 | 14.70 | 6.18 | 34.96 |
| S+ | 2017 | 3.85 | 0.25 | 58.43 | 60.65 | 21.09 | 174.46 | 3.35 | 1.00 | 11.20 |
| S+ | 2018 | 59.81 | 27.61 | 129.52 | 70.11 | 23.45 | 209.64 | 5.84 | 2.05 | 16.69 |
| W | 2015 | 18.72 | 5.64 | 62.16 | 32.75 | 11.70 | 91.64 | 2.32 | 0.34 | 15.98 |
| W | 2016 | 0.00 |  |  | 0.00 |  |  | 0.00 |  |  |
| W | 2017 | 13.27 | 2.12 | 83.09 | 0.00 |  |  | 1.07 | 0.16 | 7.06 |
| W | 2018 | 0.00 |  |  | 0.00 |  |  | 0.00 |  |  |
| P | 2015 | 181.34 | 56.65 | 580.46 | 17.86 | 5.48 | 58.25 | 12.10 | 4.26 | 34.38 |
| P | 2016 | 41.25 | 15.23 | 111.71 | 21.31 | 6.30 | 72.07 | 7.53 | 3.36 | 16.88 |
| P | 2017 | 10.48 | 2.82 | 38.96 | 0.00 |  |  | 2.51 | 0.95 | 6.62 |
| P | 2018 | 21.50 | 9.79 | 47.21 | 7.15 | 0.85 | 60.13 | 3.04 | 1.01 | 9.12 |
| SW | 2015 | 92.52 | 47.23 | 181.23 | 34.66 | 18.63 | 64.50 | 2.74 | 1.43 | 5.24 |
| SW | 2016 | 76.59 | 28.52 | 205.71 | 21.99 | 10.26 | 47.12 | 3.75 | 1.89 | 7.43 |
| SW | 2017 | 51.76 | 32.64 | 82.09 | 46.72 | 25.16 | 86.75 | 5.65 | 3.30 | 9.67 |
| SW | 2018 | 63.34 | 32.92 | 121.87 | 54.79 | 28.53 | 105.23 | 6.26 | 3.55 | 11.04 |
| Stratum | Year | T.'s gazelle S | T.' s gazelle SL | T.'s gazelle SU | Kirk's dik-dik S | Kirk's dik-dik SL | Kirk's dik-dik SU | Aardvark | Aardvark L | Aardvark U |
| W+ | 2015 | 0.18 | 0.03 | 1.10 | 3.34 | 0.75 | 14.90 | 48.18 | 20.95 | 110.81 |
| W+ | 2016 | 0.00 |  |  | 2.30 | 0.37 | 14.20 | 664.53 | 210.81 | 2094.80 |
| W+ | 2017 | 0.00 |  |  | 3.44 | 0.78 | 15.27 | 314.56 | 132.75 | 745.36 |
| W+ | 2018 | 0.00 |  |  | 4.66 | 1.83 | 11.84 | 428.02 | 173.06 | 1058.60 |
| G+ | 2015 | 6.03 | 2.13 | 17.03 | 0.00 |  |  | 36.86 | 9.90 | 137.19 |
| G+ | 2016 | 7.12 | 2.70 | 18.80 | 0.00 |  |  | 75.15 | 21.82 | 258.78 |
| G+ | 2017 | 8.12 | 2.83 | 23.33 | 0.00 |  |  | 24.89 | 7.81 | 79.30 |
| G+ | 2018 | 3.17 | 1.18 | 8.51 | 0.00 |  |  | 284.32 | 89.15 | 906.72 |
| S+ | 2015 | 0.21 | 0.03 | 1.33 | 4.01 | 0.64 | 24.99 | 115.64 | 22.49 | 594.49 |
| S+ | 2016 | 1.51 | 0.24 | 9.49 | 0.00 |  |  | 77.50 | 30.17 | 199.06 |
| S+ | 2017 | 0.11 | 0.02 | 0.67 | 0.00 |  |  | 126.34 | 41.63 | 383.41 |
| S+ | 2018 | 0.68 | 0.06 | 8.09 | 0.00 |  |  | 350.70 | 153.96 | 798.88 |
| W | 2015 | 0.62 | 0.09 | 4.23 | 0.00 |  |  | 470.13 | 241.45 | 915.40 |
| W | 2016 | 0.00 |  |  | 0.00 |  |  | 299.38 | 88.57 | 1012.00 |
| W | 2017 | 0.00 |  |  | 4.83 | 1.03 | 22.54 | 348.04 | 144.90 | 835.99 |
| W | 2018 | 0.00 |  |  | 2.70 | 0.68 | 10.73 | 359.77 | 143.81 | 900.03 |
| P | 2015 | 16.85 | 9.21 | 30.84 | 0.79 | 0.14 | 4.67 | 223.21 | 60.90 | 818.16 |
| P | 2016 | 10.33 | 5.01 | 21.27 | 1.59 | 0.42 | 6.09 | 281.76 | 117.30 | 676.83 |
| P | 2017 | 4.48 | 2.35 | 8.53 | 0.00 |  |  | 608.02 | 188.52 | 1961.00 |
| P | 2018 | 9.36 | 4.49 | 19.49 | 0.80 | 0.14 | 4.69 | 270.12 | 123.51 | 590.75 |
| SW | 2015 | 1.14 | 0.34 | 3.88 | 2.70 | 0.72 | 10.11 | 34.09 | 16.27 | 71.41 |
| SW | 2016 | 0.00 |  |  | 5.66 | 2.73 | 11.72 | 230.31 | 105.31 | 503.69 |
| SW | 2017 | 0.51 | 0.18 | 1.47 | 1.00 | 0.33 | 3.03 | 137.39 | 83.99 | 224.75 |
| SW | 2018 | 0.00 |  |  | 1.03 | 0.24 | 4.42 | 222.77 | 123.58 | 401.59 |
| Stratum | Year | Bushbuck | Bushbuck L | Bushbuck U | Bushpig | Bushpig L | Bushpig U | G.kudu | G. kudu L | G. kudu U |
| W+ | 2015 | 519.32 | 160.08 | 1684.80 | 1907.20 | 886.55 | 4103.00 | 4357.50 | 2821.00 | 6731.10 |
| W+ | 2016 | 823.78 | 209.01 | 3246.70 | 1052.30 | 501.38 | 2208.50 | 4967.10 | 2864.60 | 8612.50 |
| W+ | 2017 | 35.69 | 5.82 | 218.75 | 2162.90 | 966.82 | 4838.50 | 2204.60 | 1066.90 | 4555.50 |
| W+ | 2018 | 217.12 | 22.66 | 2080.30 | 1628.00 | 884.02 | 2998.10 | 3373.80 | 1786.10 | 6372.60 |
| G+ | 2015 | 0.00 |  |  | 0.00 |  |  | 432.17 | 158.22 | 1180.40 |
| G+ | 2016 | 0.00 |  |  | 74.38 | 18.75 | 295.07 | 31.47 | 5.40 | 183.52 |
| G+ | 2017 | 0.00 |  |  | 147.81 | 15.62 | 1399.10 | 187.61 | 48.25 | 729.56 |
| G+ | 2018 | 26.08 | 4.45 | 152.92 | 47.90 | 12.84 | 178.66 | 30.40 | 5.20 | 177.68 |
| S+ | 2015 | 0.00 |  |  | 0.00 |  |  | 290.53 | 47.03 | 1794.70 |
| S+ | 2016 | 0.00 |  |  | 0.00 |  |  | 0.00 |  |  |
| S+ | 2017 | 0.00 |  |  | 0.00 |  |  | 0.00 |  |  |
| S+ | 2018 | 37.80 | 6.05 | 236.21 | 0.00 |  |  | 0.00 |  |  |
| W | 2015 | 241.32 | 76.29 | 763.30 | 1639.60 | 845.32 | 3180.00 | 3824.70 | 1967.20 | 7436.40 |
| W | 2016 | 461.02 | 123.50 | 1720.90 | 550.26 | 196.67 | 1539.60 | 1504.40 | 734.62 | 3080.60 |
| W | 2017 | 600.26 | 153.66 | 2344.80 | 734.83 | 299.01 | 1805.90 | 2739.90 | 1703.10 | 4407.80 |
| W | 2018 | 754.65 | 277.06 | 2055.50 | 1077.80 | 394.97 | 2941.10 | 4006.40 | 1980.60 | 8104.50 |
| P | 2015 | 0.00 |  |  | 226.57 | 53.96 | 951.33 | 1351.70 | 399.89 | 4568.70 |
| P | 2016 | 99.17 | 17.06 | 576.41 | 500.80 | 210.40 | 1192.10 | 924.61 | 274.02 | 3119.90 |
| P | 2017 | 0.00 |  |  | 272.49 | 84.71 | 876.54 | 115.29 | 19.90 | 668.12 |
| P | 2018 | 0.00 |  |  | 159.26 | 14.09 | 1800.80 | 57.76 | 9.93 | 335.94 |
| SW | 2015 | 73.49 | 24.71 | 218.51 | 144.58 | 62.94 | 332.11 | 966.50 | 523.11 | 1785.70 |
| SW | 2016 | 51.72 | 12.30 | 217.50 | 322.90 | 135.71 | 768.33 | 759.46 | 396.91 | 1453.10 |
| SW | 2017 | 83.14 | 21.38 | 323.31 | 400.77 | 197.59 | 812.89 | 399.69 | 218.68 | 730.51 |
| SW | 2018 | 533.63 | 271.30 | 1049.60 | 656.52 | 254.18 | 1695.80 | 1119.40 | 606.46 | 2066.20 |
| Stratum | Year | Lesser kudu | Lesser kudu L | Lesser kuduU | Hyena | Hyena L | Hyena U | Impala | Impala L | Impala U |
| W+ | 2015 | 576.19 | 228.51 | 1452.90 | 65.41 | 26.44 | 161.84 | 1568.40 | 776.55 | 3167.60 |
| W+ | 2016 | 1087.00 | 315.34 | 3746.70 | 422.91 | 188.98 | 946.43 | 3930.50 | 1766.20 | 8747.20 |
| W+ | 2017 | 1362.70 | 499.11 | 3720.80 | 387.74 | 129.02 | 1165.30 | 7196.30 | 4175.80 | 12402.00 |
| W+ | 2018 | 1169.10 | 520.73 | 2624.60 | 188.01 | 71.82 | 492.15 | 3255.50 | 1992.40 | 5319.30 |
| G+ | 2015 | 544.56 | 156.62 | 1893.40 | 112.60 | 41.68 | 304.15 | 1537.90 | 577.15 | 4098.10 |
| G+ | 2016 | 422.96 | 63.22 | 2829.80 | 89.28 | 32.12 | 248.16 | 1567.80 | 504.68 | 4870.40 |
| G+ | 2017 | 52.54 | 9.00 | 306.67 | 152.07 | 56.53 | 409.09 | 1780.40 | 690.89 | 4588.00 |
| G+ | 2018 | 25.54 | 4.37 | 149.34 | 98.56 | 44.96 | 216.06 | 4260.30 | 1580.30 | 11485.00 |
| S+ | 2015 | 0.00 |  |  | 137.37 | 58.99 | 319.89 | 106.04 | 34.31 | 327.76 |
| S+ | 2016 | 40.89 | 6.62 | 252.41 | 197.28 | 27.52 | 1414.40 | 133.25 | 20.23 | 877.76 |
| S+ | 2017 | 41.02 | 6.65 | 253.15 | 79.17 | 21.06 | 297.64 | 80.21 | 12.33 | 521.74 |
| S+ | 2018 | 0.00 |  |  | 142.84 | 50.35 | 405.25 | 458.28 | 126.10 | 1665.50 |
| W | 2015 | 2645.90 | 1429.40 | 4897.90 | 113.98 | 14.43 | 900.45 | 554.29 | 161.61 | 1901.10 |
| W | 2016 | 1715.00 | 417.21 | 7050.10 | 130.65 | 35.30 | 483.49 | 58.83 | 15.37 | 225.15 |
| W | 2017 | 2742.30 | 1477.20 | 5091.00 | 354.39 | 132.44 | 948.28 | 2361.70 | 1127.70 | 4946.40 |
| W | 2018 | 738.78 | 371.72 | 1468.30 | 217.82 | 87.27 | 543.65 | 588.49 | 221.52 | 1563.40 |
| P | 2015 | 628.12 | 253.42 | 1556.90 | 384.63 | 148.16 | 998.52 | 3763.10 | 1799.50 | 7868.90 |
| P | 2016 | 145.63 | 55.01 | 385.55 | 117.10 | 50.36 | 272.31 | 4951.40 | 2315.40 | 10588.00 |
| P | 2017 | 169.49 | 41.66 | 689.57 | 46.73 | 18.67 | 116.94 | 3471.60 | 1664.40 | 7241.20 |
| P | 2018 | 509.45 | 144.42 | 1797.20 | 152.16 | 73.82 | 313.62 | 5201.80 | 2139.70 | 12646.00 |
| SW | 2015 | 369.98 | 170.95 | 800.71 | 114.04 | 66.60 | 195.27 | 2672.50 | 1693.80 | 4216.70 |
| SW | 2016 | 344.30 | 147.36 | 804.47 | 151.45 | 88.41 | 259.46 | 3662.90 | 2183.20 | 6145.40 |
| SW | 2017 | 295.06 | 151.78 | 573.60 | 166.90 | 102.55 | 271.63 | 4091.30 | 2638.20 | 6344.90 |
| SW | 2018 | 564.20 | 319.25 | 997.10 | 191.55 | 104.66 | 350.57 | 5216.00 | 3167.40 | 8589.80 |
| Stratum | Year | Zebra | Zebra L | Zebra U | Kirk's dik dik | Kirk's dik dik L | Kirk's dik dik U | Eland | Eland L | Eland U |
| W+ | 2015 | 954.00 | 299.92 | 3034.50 | 4859.30 | 2779.30 | 8495.80 | 1309.30 | 561.96 | 3050.40 |
| W+ | 2016 | 592.15 | 239.10 | 1466.50 | 4072.10 | 2370.30 | 6995.70 | 1354.50 | 747.50 | 2454.30 |
| W+ | 2017 | 0.00 |  |  | 7416.50 | 5367.10 | 10248.00 | 2051.70 | 993.33 | 4237.60 |
| W+ | 2018 | 697.99 | 235.53 | 2068.50 | 9164.90 | 6628.60 | 12672.00 | 2052.70 | 787.29 | 5352.00 |
| G+ | 2015 | 170.31 | 42.73 | 678.75 | 726.93 | 236.08 | 2238.40 | 100.17 | 29.09 | 344.95 |
| G+ | 2016 | 223.22 | 45.64 | 1091.60 | 804.57 | 275.38 | 2350.70 | 857.77 | 238.29 | 3087.60 |
| G+ | 2017 | 0.00 |  |  | 631.16 | 194.35 | 2049.70 | 1846.70 | 847.38 | 4024.60 |
| G+ | 2018 | 0.00 |  |  | 654.46 | 221.47 | 1933.90 | 335.37 | 90.54 | 1242.30 |
| S+ | 2015 | 114.49 | 17.56 | 746.34 | 162.90 | 42.14 | 629.79 | 31.43 | 5.09 | 194.17 |
| S+ | 2016 | 0.00 |  |  | 556.77 | 160.41 | 1932.50 | 0.00 |  |  |
| S+ | 2017 | 0.00 |  |  | 32.86 | 5.33 | 202.66 | 95.08 | 22.62 | 399.72 |
| S+ | 2018 | 0.00 |  |  | 444.63 | 124.09 | 1593.10 | 142.96 | 21.49 | 950.94 |
| W | 2015 | 576.29 | 88.21 | 3765.00 | 4730.50 | 2791.80 | 8015.40 | 2299.70 | 610.44 | 8663.80 |
| W | 2016 | 465.79 | 115.42 | 1879.80 | 6470.60 | 3673.60 | 11397.00 | 2371.10 | 781.02 | 7198.30 |
| W | 2017 | 367.56 | 136.49 | 989.82 | 5216.60 | 3240.30 | 8398.10 | 983.67 | 546.12 | 1771.80 |
| W | 2018 | 885.68 | 414.50 | 1892.50 | 3155.90 | 1855.90 | 5366.40 | 1014.70 | 349.48 | 2946.10 |
| P | 2015 | 906.65 | 346.08 | 2375.20 | 3850.60 | 1379.80 | 10746.00 | 765.24 | 263.35 | 2223.70 |
| P | 2016 | 0.00 |  |  | 1322.00 | 588.92 | 2967.50 | 1087.60 | 361.08 | 3276.10 |
| P | 2017 | 0.00 |  |  | 620.57 | 263.72 | 1460.30 | 37.41 | 6.43 | 217.70 |
| P | 2018 | 45.52 | 7.83 | 264.81 | 524.63 | 210.49 | 1307.60 | 18.74 | 3.23 | 108.69 |
| SW | 2015 | 414.62 | 214.80 | 800.34 | 781.99 | 453.61 | 1348.10 | 317.60 | 119.73 | 842.44 |
| SW | 2016 | 161.52 | 47.03 | 554.76 | 2303.50 | 1659.80 | 3196.80 | 430.30 | 207.27 | 893.31 |
| SW | 2017 | 28.64 | 5.33 | 153.80 | 1605.40 | 1114.50 | 2312.40 | 581.68 | 311.56 | 1086.00 |
| SW | 2018 | 117.63 | 34.83 | 397.33 | 2284.60 | 1433.80 | 3640.20 | 548.90 | 272.78 | 1104.50 |
| Stratum | Year | Elephant | Elephant L | Elephant U | Giraffe | Giraffe L | Giraffe U | Warthog | Warthog L | Warthog U |
| W+ | 2015 | 211.57 | 48.80 | 917.32 | 591.82 | 353.14 | 991.82 | 254.86 | 107.54 | 603.97 |
| W+ | 2016 | 117.25 | 25.26 | 544.23 | 730.74 | 352.00 | 1517.00 | 444.92 | 199.15 | 993.99 |
| W+ | 2017 | 171.38 | 88.80 | 330.76 | 1338.30 | 629.09 | 2847.20 | 180.64 | 63.82 | 511.31 |
| W+ | 2018 | 236.93 | 88.81 | 632.12 | 3212.60 | 1762.60 | 5855.50 | 899.04 | 234.65 | 3444.50 |
| G+ | 2015 | 23.12 | 5.50 | 97.20 | 160.67 | 44.79 | 576.29 | 24.37 | 6.53 | 90.97 |
| G+ | 2016 | 70.72 | 11.35 | 440.78 | 29.78 | 8.02 | 110.56 | 12.42 | 2.13 | 72.57 |
| G+ | 2017 | 0.00 |  |  | 29.59 | 5.07 | 172.65 | 0.00 |  |  |
| G+ | 2018 | 0.00 |  |  | 43.15 | 10.84 | 171.77 | 0.00 |  |  |
| S+ | 2015 | 0.00 |  |  | 0.00 |  |  | 0.00 |  |  |
| S+ | 2016 | 27.35 | 4.42 | 169.19 | 0.00 |  |  | 0.00 |  |  |
| S+ | 2017 | 0.00 |  |  | 69.32 | 11.25 | 427.05 | 0.00 |  |  |
| S+ | 2018 | 0.00 |  |  | 0.00 |  |  | 0.00 |  |  |
| W | 2015 | 10.53 | 1.68 | 66.06 | 212.91 | 64.47 | 703.10 | 199.85 | 60.00 | 665.65 |
| W | 2016 | 0.00 |  |  | 76.26 | 11.47 | 507.18 | 106.05 | 13.22 | 851.05 |
| W | 2017 | 0.00 |  |  | 55.17 | 13.55 | 224.54 | 276.17 | 127.10 | 600.07 |
| W | 2018 | 0.00 |  |  | 0.00 |  |  | 385.78 | 88.42 | 1683.10 |
| P | 2015 | 0.00 |  |  | 108.86 | 34.53 | 343.20 | 11.35 | 1.96 | 65.92 |
| P | 2016 | 27.06 | 4.66 | 157.25 | 560.53 | 200.98 | 1563.30 | 0.00 |  |  |
| P | 2017 | 0.00 |  |  | 572.79 | 186.07 | 1763.20 | 68.27 | 15.99 | 291.52 |
| P | 2018 | 0.00 |  |  | 1339.10 | 483.85 | 3706.20 | 57.01 | 13.95 | 232.93 |
| SW | 2015 | 11.46 | 3.51 | 37.37 | 191.03 | 95.85 | 380.70 | 82.11 | 35.42 | 190.33 |
| SW | 2016 | 6.77 | 1.78 | 25.81 | 313.71 | 165.65 | 594.13 | 99.94 | 39.99 | 249.77 |
| SW | 2017 | 11.34 | 3.02 | 42.66 | 366.77 | 188.34 | 714.25 | 38.25 | 13.83 | 105.79 |
| SW | 2018 | 2.33 | 0.43 | 12.53 | 841.56 | 516.91 | 1370.10 | 137.49 | 40.80 | 463.29 |
| Stratum | Year | Wildebeest | Wildebeest L | Wildebeest U |  |  |  |  |  |  |
| W+ | 2015 | 345.10 | 76.25 | 1561.90 |  |  |  |  |  |  |
| W+ | 2016 | 0.00 |  |  |  |  |  |  |  |  |
| W+ | 2017 | 0.00 |  |  |  |  |  |  |  |  |
| W+ | 2018 | 0.00 |  |  |  |  |  |  |  |  |
| G+ | 2015 | 1518.20 | 683.96 | 3369.90 |  |  |  |  |  |  |
| G+ | 2016 | 592.15 | 127.63 | 2747.40 |  |  |  |  |  |  |
| G+ | 2017 | 9427.70 | 5378.00 | 16527.00 |  |  |  |  |  |  |
| G+ | 2018 | 3652.90 | 1878.80 | 7102.20 |  |  |  |  |  |  |
| S+ | 2015 | 331.34 | 71.26 | 1540.70 |  |  |  |  |  |  |
| S+ | 2016 | 0.00 |  |  |  |  |  |  |  |  |
| S+ | 2017 | 125.31 | 20.34 | 772.19 |  |  |  |  |  |  |
| S+ | 2018 | 0.00 |  |  |  |  |  |  |  |  |
| W | 2015 | 0.00 |  |  |  |  |  |  |  |  |
| W | 2016 | 0.00 |  |  |  |  |  |  |  |  |
| W | 2017 | 0.00 |  |  |  |  |  |  |  |  |
| W | 2018 | 0.00 |  |  |  |  |  |  |  |  |
| P | 2015 | 184.49 | 65.20 | 522.04 |  |  |  |  |  |  |
| P | 2016 | 12.36 | 2.13 | 71.63 |  |  |  |  |  |  |
| P | 2017 | 0.00 |  |  |  |  |  |  |  |  |
| P | 2018 | 0.00 |  |  |  |  |  |  |  |  |
| SW | 2015 | 266.84 | 92.22 | 772.12 |  |  |  |  |  |  |
| SW | 2016 | 67.02 | 19.93 | 225.36 |  |  |  |  |  |  |
| SW | 2017 | 165.75 | 47.17 | 582.50 |  |  |  |  |  |  |
| SW | 2018 | 95.75 | 34.21 | 267.94 |  |  |  |  |  |  |
