# Supplementary material for: Land use, REDD+ and the status of wildlife populations in Yaeda Valley, northern Tanzania
Source: PLoS One. 2019 Apr 4;14(4):e0214823. doi: 10.1371/journal.pone.0214823 (PMC6448838; doi:10.1371/journal.pone.0214823)
Supplement: S4 Data — Population growth rates were estimated from 2015 to 2018 for all six strata [three REDD+ strata (W+, G+, and S+) three control land-use strata (W, P, and SW)] using generalized linear models with log-link. Empty cells denote that the species were not detected in this stratum. Regression coefficients for species highlighted in grey were not utilized for comparisons across land-use strata (main manuscript Fig 6) because those species were either livestock species, or had only one estimate in time that exceeded 0 signs or individuals per km2. For Kirk’s dik-dik we used the sign density, because signs were observed in all strata. (DOCX) [file pone.0214823.s004.docx]

**S 4 Data. Regression coefficient estimates (β) and associated p-values for the effect of year on population density estimates of livestock and wildlife species in Yaeda Valley, Tanzania.** Population growth rates were estimated from 2015 to 2018 for all six strata [three Redd+ strata (W+, G+, and S+) three control land-use strata (W, P, and SW)] using generalized linear models with log-link. Empty cells denote that the species were not detected in this stratum. Regression coefficients for species highlighted in grey were not utilized for comparisons across land-use strata (main manuscript Fig. 6) because those species were either livestock species, or had only one estimate in time that exceeded 0 signs or individuals per km². For Kirk’s dik-dik we used the sign density, because signs were observed in all strata.

|  | **W+** | | **G+** | | **S+** | | **W** | | **P** | | **SW** | |
| --- | --- | --- | --- | --- | --- | --- | --- | --- | --- | --- | --- | --- |
| **Species** | **β** | **p-value** | **β** | **p-value** | **β** | **p-value** | **β** | **p-value** | **β** | **p-value** | **β** | **p-value** |
| Cattle - sightings | 0.129 | 0.808 | -0.806 | 0.116 | -0.863 | 0.087 | -0.771 | 0.454 | -1.403 | 0.037 | -0.017 | 0.141 |
| Donkey - sightings |  |  | -0.394 | 0.316 | -0.806 | 0.038 | -2.619 | 0.624 | -0.578 | 0.039 | 0.262 | 0.040 |
| Goat & sheep sightings | -0.204 | 0.869 | -0.053 | 0.763 | -0.393 | 0.047 | -8.093 | 0.015 | -0.447 | 0.375 | 0.237 | 0.200 |
|  |  |  |  |  |  |  |  |  |  |  |  |  |
| Impala -signs | 0.157 | 0.658 | 0.472 | 0.103 | 0.956 | 0.178 | 0.200 | 0.777 | 0.067 | 0.564 | 0.207 | 0.014 |
| Wildebeest - signs | -10.449 | 0.009 | 0.299 | 0.643 | -6.586 | 0.981 |  |  | -2.712 | 0.000 | -0.351 | 0.337 |
| Plain's zebra - signs | -0.377 | 0.437 | -0.573 | 0.402 | -9.345 | 0.011 | 0.187 | 0.426 | -11.410 | 0.998 | -0.870 | 0.109 |
| Thomson's gazelle - sightings | -2.764 | 0.084 | -0.104 | 0.616 | 0.001 | 0.999 | -4.101 | 0.051 | -0.350 | 0.200 | -2.445 | 0.574 |
| Maasai giraffe - signs | 0.736 | 0.019 | -1.141 | 0.173 | 0.214 | 0.863 | -0.902 | 0.034 | 0.631 | 0.064 | 0.561 | 0.041 |
| Hyena - signs | 0.088 | 0.821 | 0.017 | 0.909 | -0.073 | 0.730 | 0.222 | 0.470 | -0.807 | 0.198 | 0.156 | 0.026 |
| Elephant - signs | 0.085 | 0.619 | -0.381 | 0.667 | -0.204 | 0.870 | -6.958 | 0.020 | -0.204 | 0.870 | -0.260 | 0.398 |
| Kirk's dik dik - sightings | 0.169 | 0.259 |  |  | -5.989 | 0.027 | 0.485 | 0.518 | -0.189 | 0.708 | -0.288 | 0.569 |
| Kirk's dik dik - signs | 0.273 | 0.081 | -0.055 | 0.365 | 0.111 | 0.826 | -0.105 | 0.509 | -0.940 | 0.019 | 0.200 | 0.377 |
| Aardvark -signs | 0.172 | 0.688 | 1.665 | 0.236 | 0.668 | 0.113 | -0.085 | 0.450 | 0.109 | 0.725 | 0.268 | 0.424 |
| Warthog - signs | 0.532 | 0.261 | -0.987 | 0.081 |  |  | 0.353 | 0.156 | 0.527 | 0.354 | 0.145 | 0.609 |
| Bushpig - signs | 0.017 | 0.923 | 0.207 | 0.708 |  |  | -0.213 | 0.473 | -0.116 | 0.706 | 0.435 | 0.020 |
| Lesser kudu-signs | 0.172 | 0.293 | -0.688 | 0.143 | 0.000 | 1.000 | -0.217 | 0.421 | -0.170 | 0.691 | 0.160 | 0.346 |
| Eland -signs | 0.171 | 0.105 | 0.135 | 0.824 | 0.724 | 0.148 | -0.304 | 0.163 | -0.525 | 0.423 | 0.168 | 0.137 |
| Bushbuck - signs | -0.369 | 0.481 | 7.866 | 0.016 | 8.237 | 0.015 | 0.314 | 0.033 | -0.217 | 0.861 | 1.637 | 0.085 |
| Greater kudu - signs | -0.156 | 0.390 | -1.185 | 0.302 | -10.277 | 0.009 | 0.077 | 0.743 | -0.748 | 0.104 | 0.016 | 0.947 |
