# Supplementary figures and images for: Land use, REDD+ and the status of wildlife populations in Yaeda Valley, northern Tanzania
Source: PLoS One. 2019 Apr 4;14(4):e0214823. doi: 10.1371/journal.pone.0214823 (PMC6448838; doi:10.1371/journal.pone.0214823)

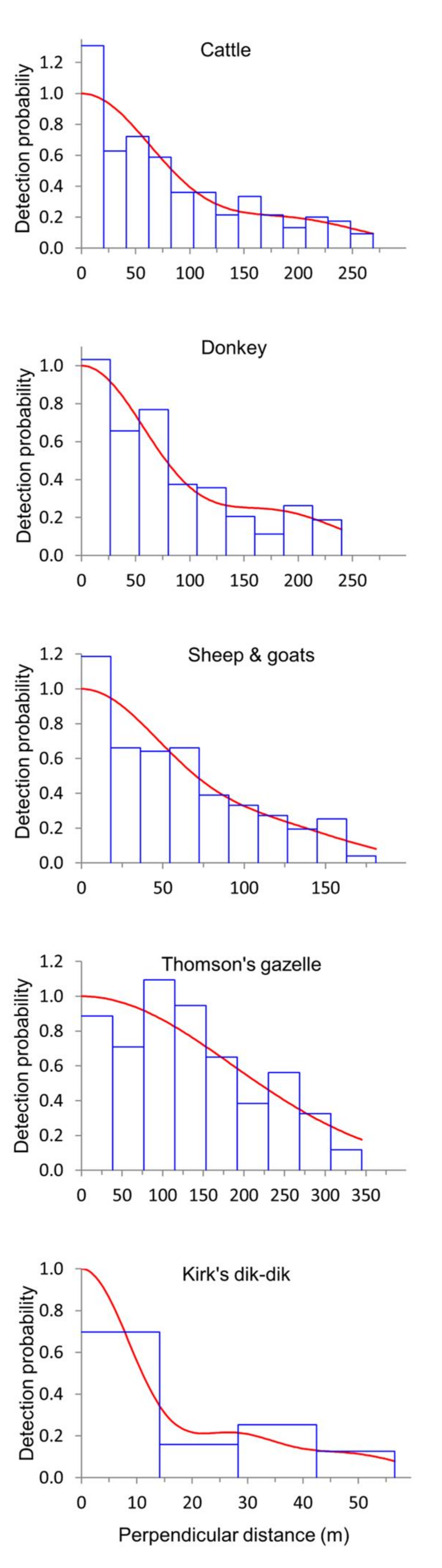

Supplement: S1 Fig — Histograms (blue bars) represent sighting frequency and the red line is the fitted detection function. (TIFF) [file pone.0214823.s005.tiff]

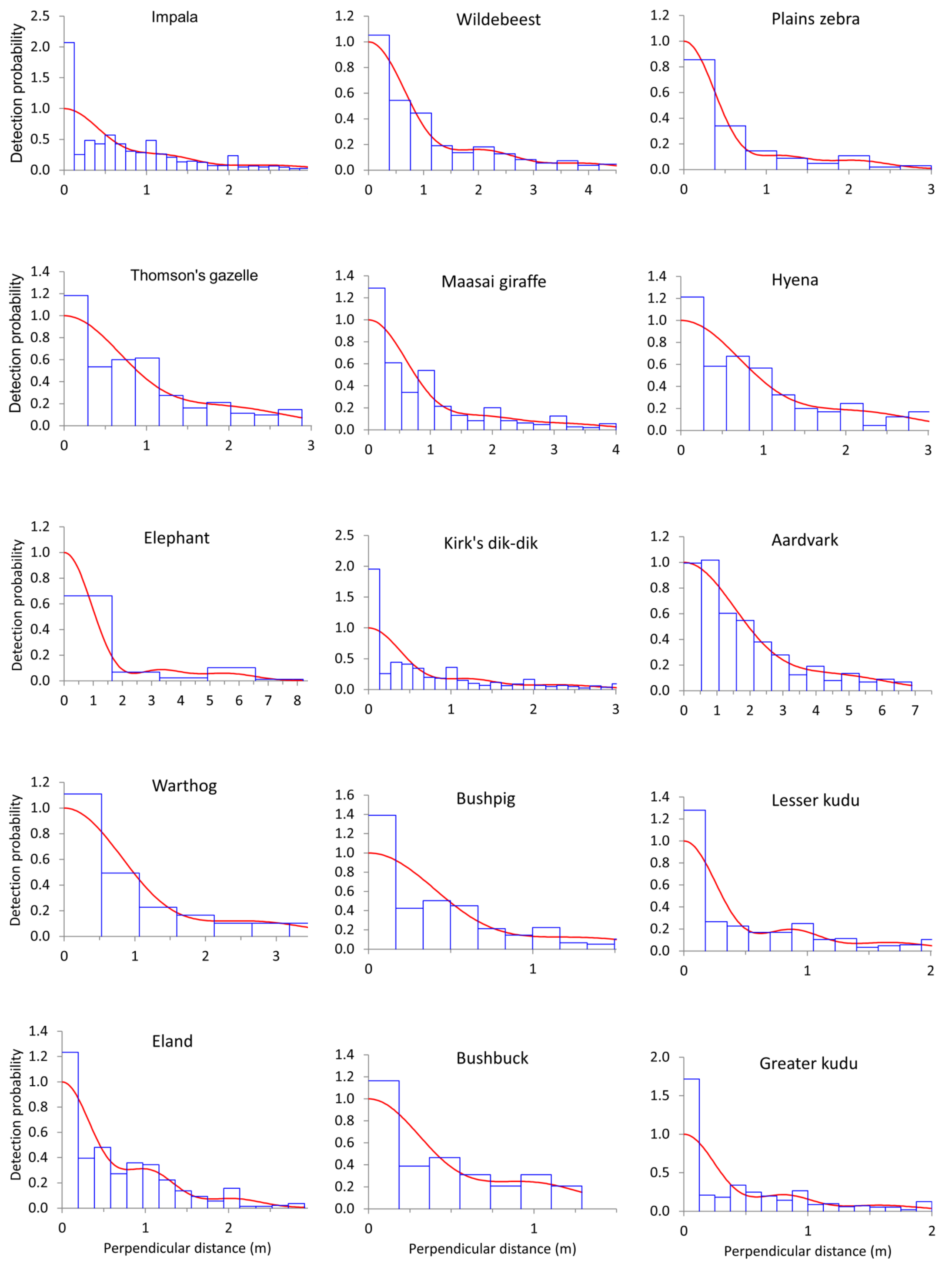

Supplement: S2 Fig — Histograms (blue bars) represent sighting frequency and the red line is the fitted detection function. (TIFF) [file pone.0214823.s006.tiff]
